# New Coleoptera records from New Brunswick, Canada: Anthribidae, Brentidae, Dryophthoridae, Brachyceridae, and Curculionidae, with additions to the fauna of Quebec, Nova Scotia and Prince Edward Island

**DOI:** 10.3897/zookeys.179.2626

**Published:** 2012-04-04

**Authors:** Reginald P. Webster, Robert S. Anderson, Jon D. Sweeney, Ian DeMerchant

**Affiliations:** 1 Natural Resources Canada, Canadian Forest Service - Atlantic Forestry Centre, 1350 Regent St., P.O. Box 4000, Fredericton, NB, Canada E3B 5P7; 2 Research Division, Canadian Museum of Nature, P.O. Box 3443, Station D, Ottawa, ON, Canada K1P6P4

**Keywords:** Anthribidae, Brachyceridae, Brentidae, Dryophthoridae, Curculionidae, new records, Canada, New Brunswick, Nova Scotia, Prince Edward Island, weevils, bark beetles, Maritime provinces

## Abstract

We report 63 species of Curculionoidea that are new to New Brunswick (three species of Anthribidae, four species of Brentidae, three species of Dryophthoridae, three species of Brachyceridae, 50 species of Curculionidae). Among these are 27 species (two Anthribidae, two Brenthidae, one Brachyceridae, 22 Curculionidae) that are also newly recorded for the Maritime provinces, and one species, *Plesiobaris disjuncta* Casey (Curculionidae) that is newly recorded for Canada from New Brunswick and Quebec. *Bagous planatus* LeConte is reinstated to the faunal list of New Brunswick. Two species of Curculionidae are newly recorded from Nova Scotia and the Maritime provinces, and two others are reported for the first time for Prince Edward Island.

## Introduction

The Curculioniodea, or weevils, occurring in New Brunswick include the families Nemonychidae (the pine flower snout beetles), Anthribidae (the fungus weevils), Attelabidae (the leaf-rolling weevils), Brentidae (the straight-snouted weevils and pear-shaped weevils), and the Dryophthoridae, Brachyceridae, and Curculionidae, previously, all considered members of the Curculionidae (weevils or snout beetles and bark beetles) by Anderson (2002). The classification used here follows the synthesis of Bouchard et al. (2011), based on changes proposed by Thompson (1992), Kuschel (1995), Lawrence and Newton (1995), and Alonso-Zarazaga and Lyal (1999). The weevils of New Brunswick were reviewed by Majka et al. (2007b). They reported 77 new species records for the province, increasing the weevil fauna to 206 species. Three of these species, *Trichapion nigrum* (Herbst), *Ceutorhynchus semirufus* LeConte, and *Listronotus laramienis* (Angell), were recorded for the first time from Canada. *Bagous planatus* LeConte, *Plocamus hispidulus* LeConte, and *Dryocoetes granicollis* (LeConte) were removed from the faunal list of New Brunswick due to a lack of supporting voucher specimens or other published records (Majka et al. 2007b). More recently the brentid, *Arrenodes minutus* (Drury) was reported from New Brunswick by Majka et al. (2008). Other Curculionoidea from Nova Scotia and Prince Edward Island were reported by Bright and Bouchard (2008), Majka (2010b), and Klimaszewski et al. (2010). Here, we report 63 species of Curculionoidea that are new to New Brunswick, two species new from Nova Scotia and two species new for Prince Edward Island.

## Methods and conventions

The following records are based in part on specimens collected as part of a general survey by the first author to document the Coleoptera fauna of New Brunswick. A description of the habitat was recorded for all specimens collected during this survey and was included on specimen labels. This information is included with each record and summarized in the collection and habitat data section for each species.

### Collection methods

Various collection methods were employed to collect the specimens reported in this study. Details are outlined in Webster et al. (2009, Appendix). Some specimens were collected from Lindgren funnel trap samples during a study to develop a general attractant to detect invasive species of Cerambycidae. These traps visually mimic tree trunks and are often effective for sampling species of Coleoptera that live in microhabitats associated with standing trees (Lindgren 1983). See Webster et al. (in press) for details of the methods used to deploy Lindgren 12-funnel traps and for sample collection. New records were also obtained from the insect collection belonging to Natural Resources Canada, Canadian Forest Service - Atlantic Canada Forestry Centre, Fredericton.

### Distribution

Distribution maps, created using ArcMap and ArcGIS, are presented for each species in New Brunswick. Every species is cited with current distribution in Canada and Alaska, using abbreviations for the state, provinces, and territories. New provincial records are indicated in bold under Distribution in Canada and Alaska. The following abbreviations are used in the text:

**Table:** 

**AK**	Alaska	**MB**	Manitoba
**YT**	Yukon Territory	**ON**	Ontario
**NT**	Northwest Territories	**QC**	Quebec
**NU**	Nunavut	**NB**	New Brunswick
**BC**	British Columbia	**PE**	Prince Edward Island
**AB**	Alberta	**NS**	Nova Scotia
**SK**	Saskatchewan	**NF & LB**	Newfoundland and Labrador*

* Newfoundland and Labrador are each treated separately under the current Distribution in Canada and Alaska.

Acronyms of collections examined and referred to in this study are as follows:

**AFC** Atlantic Forestry Centre, Natural Resources Canada, Canadian Forest Service, Fredericton, New Brunswick, Canada

**CCC** Claude Chantal Collection, Varennes, Quebec, Canada

**CNC** Canadian National Collection of Insects, Arachnids and Nematodes, Agriculture and Agri-Food Canada, Ottawa, Ontario, Canada

**NBM** New Brunswick Museum, Saint John, New Brunswick, Canada

**RWC** Reginald P. Webster Collection, Charters Settlement, New Brunswick, Canada

## Results

We report 63 species of Curculionoidea new to New Brunswick (three species of Anthribidae, four species of Brentidae, three species of Dryophthoridae, three species of Brachyceridae, 50 species of Curculionidae). Among these are 27 species (two Anthribidae, two Brenthidae, one Brachyceridae, 22 Curculionidae) that are also newly recorded for the Maritime provinces (New Brunswick, Nova Scotia, Prince Edward Island), and one species newly recorded for Canada (Table 1). Two species of Curculionidae are newly recorded for Nova Scotia and two for Prince Edward Island.

**Table 1.** Species of Nemonychidae, Anthribidae, Attelabidae, Brentidae, Dryophthoridae, Brachyceridae, and Curculionidae (Curculionoidea) recorded from New Brunswick, Prince Edward Island, and Nova Scotia, Canada.

**Species**	**NB**	**NS**	**PE**
**Family Nemonychidae Bedel**			
**Subfamily Cimberidinae Gozis**			
**Tribe Cimberidini Gozis**			
*Cimberis elongata* (LeConte)	X	X	
*Cimberis pallipennis* (Blatchley)		X	
*Cimberis pilosa* (LeConte)	X		
**Family Anthribidae Billberg**			
**Subfamily Anthribinae Billberg**			
**Tribe Cratoparini LeConte**			
*Euparius marmoreus* (Olivier)	X	X	
**Tribe Stenocerini Kolbe**			
*Allandrus bifasciatus* LeConte	X	X	
*Allandrus populi* Pierce		X	
**Tribe Tropiderini Lacordaire**			
*Eurymycter fasciatus* (Olivier)	X	X	
*Eurymycter latifascia* Pierce	X*	X	
**Tribe Trigonorhinini Valentine**			
*Trigonorhinus limbatus* (Say)		X	
*Trigonorhinus sticticus* (Boheman)	X		X
**Tribe Zygaenodini Lacordaire**			
*Ormiscus saltator* (LeConte)	X**		
**Subfamily Choraginae Kirby**			
**Tribe Choragini Kirby**			
*Choragus sayi* LeConte	X**		
**Family Attelabidae Billberg**			
**Subfamily Attelabinae Billberg**			
**Tribe Attelabini Billberg**			
*Attelabus bipustulatus* Fabricius		X	
*Himatolabus pubescens* (Say)	X	X	
**Subfamily Rhynchitinae Gistel**			
**Tribe Auletini Desbrochers des Loges**			
*Auletobius cassandrae* (LeConte)	X	X	X
**Tribe Rhynchitini Gistel**			
*Temnocerus cyanellus* (LeConte)	X	X	
*Temnocerus perplexus* (Blatchley)	X	X	X
**Family Brentidae Billberg**			
**Subfamily Brentinae Billberg, 1820**			
**Tribe Brentini Billberg, 1820**			
*Arrenodes minutus* (Drury)	X	X	
**Subfamily Apioninae Schönherr, 1823**			
**Tribe Apionini Schönherr, 1823**			
*Betulapion simile simile* (Kirby) ^$^	X	X	
*Coelocephalapion carinatum* (Smith)		X	
*Coelocephalapion emaciipes* (Fall)	X*	X	
*Eutrichapion cyanitinctum* (Fall)	X	X	X
*Fallapion finitimum* Fall	X		
*Fallapion pennsylvanicum* (Boheman)	X	X	
*Neapion frosti* (Kissinger)	X*	X	
*Omphalapion hookerorum* (Kirby)^$^		X	
*Perapion curtirostre* (Germar) ^$^	X	X	X
*Podapion gallicola* Riley	X**		
*Rhopalapion longirostre* (Olivier) ^$^		X	
*Trichapion centrale* Fall		X	
*Trichapion nigrum* (Herbst)	X		
*Trichapion porcatum* (Boheman)	X**		
*Trichapion reconditum* (Gyllenhal)	X		
**Family Dryophthoridae Schönherr**			
**Subfamily Dryophthorinae Schönherr**			
**Dryophthorini Schönherr**			
*Dryophthorus americanus* Bedel	X	X	
**Subfamily Rhynchophorinae Schönherr**			
**Tribe Rhynchophorini Schönherr**			
*Sitophilus granarius* (Linnaeus) ^$^	X	X	X
*Sitophilus oryzae* (Linnaeus) ^$^	X*	X	X
**Tribe Sphenophorini Lacordaire**			
*Sphenophorus aequalis* Gyllenhal		X	
*Sphenophorus cariosus* (Olivier)		X	
*Sphenophorus costipennis* Horn	X	X	X
*Sphenophorus parvulus* (Gyllenhal)	X*	X	
*Sphenophorus pertinax* (Olivier)	X	X	
*Sphenophorus striatipennis* Chittenden	X	X	
*Sphenophorus venatus* (Say)	X	X	
*Sphenophorus zeae* Walsh	X*	X	X
**Family Brachyceridae Billberg, 1820**			
**Subfamily Erirhininae Schönherr**			
**Tribe Erirhinini Schönherr**			
*Grypus equiseti* (Fabricius)	X		
*Notaris aethiops* (Fabricius)	X	X	
*Notaris puncticollis* (LeConte)	X	X	X
*Tournotaris bimaculatus* (Fabricius)	X	X	
**Tribe Stenopelmini LeConte**			
*Notiodes ovalis* (LeConte)	X**		
*Onychylis nigrirostris* (Boheman)	X*	X	
**Tribe Tanysphyrini Gistel**			
*Tanysphyrus lemnae* (Fabricius)	X*	X	
**Family Curculionidae Latreille**			
**Subfamily Curculioninae Latreille**			
**Tribe Acalyptini Thomson**			
*Acalyptus carpini* (Herbst)	X	X	
**Tribe Anthonomini Thomson**			
*Anthonomopsis mixta* (LeConte)		X	
*Anthonomus corvulus* LeConte	X	X	X
*Anthonomus elongatus* LeConte	X	X	X
*Anthonomus haematopus* Boheman	X*	X	X
*Anthonomus interstitialus* Dietz	X		
*Anthonomus lecontei* Burke	X	X	X
*Anthonomus molochinus* Dietz	X	X	X
*Anthonomus musculus* Say		X	
*Anthonomus pictus* Blatchley		X	
*Anthonomus profundus* LeConte	X	X	
*Anthonomus quadrigibbus* (Say)	X	X	
*Anthonomus robustulus* LeConte	X		
*Anthonomus rutilus* (Boheman)	X		
*Anthonomus signatus* Say	X	X	X
*Anthonomus simiolus* Blatchely	X		
*Anthonomus subfasciatus* LeConte	X*	X	
*Pseuanthonomus crataegi* (Walsh)	X	X	
*Pseuanthonomus seriesetosus* Dietz		X	
*Pseuanthonomus validus* Dietz	X	X	X
**Tribe Curculionini Latreille**			
*Curculio iowensis* (Casey)		X	
*Curculio nascius* (Say)	X	X	
*Curculio obtusus* (Blanchard)	X**		
**Tribe Ellescini Thomson**			
*Ellescus ephippiatus* (Say)	X**		
*Dorytomus frosti* Blatchely	X**		
*Dorytomus laticollis* LeConte	X*	X	
*Dorytomus luridus* (Mannerheim)	X*	X	
*Dorytomus marmoreus* Casey	X*	X	
*Dorytomus parvicollis* Casey	X	X	
*Dorytomus rufulus* (Mannerheim)		X	
*Dorytomus vagenotatus* Casey	X		
*Proctorus armatus* LeConte	X		
*Proctorus brevicollis* LeConte		X	
*Proctorus decipiens* (LeConte)	X	X	
**Tribe Mecinini Gistel**			
*Cleopomiarus hispidulus* (LeConte)	X**		
*Mecinus janthinus* (Germar) ^$^		X	
*Mecinus pascuorum* (Gyllenhal) ^$^		X	X
*Rhinus antirrhini* (Paykull)	X	X	X
*Rhinus tetrum* (Fabricius)	X	X	X
**Tribe Piazorhinini Lacordaire**			
*Piazorhinus pictus* LeConte	X*	X	
*Piazorhinus scutellaris* (Say)	X	X	
**Tribe Rhamphini Rafinesque**			
*Isochus sequensi* (Stierlin) ^$^	X	X	X
*Isochus rufipes* (LeConte)	X	X	
*Orchestes mixtus* (Blatchley)	X	X	X
*Orchestes pallicornis* (Say)	X	X	X
*Orchestes testaceus* (Muller)	X	X	
*Tachyerges ephippiatus* (Say)	X	X	
*Tachyerges niger* (Horn)	X		
*Tachyerges salicis* (Linnaeus)	X	X	
**Tribe Smicronychini Seidlitz**			
*Smicronyx corniculatus* (Fahraeus)	X		
**Tribe Tychiini Gistel**			
*Lignyodes helvolus* (LeConte)	X		
*Tychius meliloti* Stephens^$^	X	X	X
*Tychius picirostris* (Fabricius) ^$^	X	X	X
*Tychius stephensi* Schönherr^$^	X	X	X
**Subfamily Bagoinae Thomson**			
*Bagous americanus* LeConte		X	
*Bagous nebulosus* LeConte	X		
*Bagous obliquus* LeConte	X**		
*Bagous planatus* LeConte	X**		
*Bagous restrictus* LeConte		X	
*Bagous transversus* LeConte		X	
**Subfamily Baridinae Schönherr**			
**Tribe Apostasimerini Schönherr**			
*Cylindridia prolixa* (LeConte)	X*	X	
*Dirabius rectirostris* (LeConte)	X	X	X
*Odontocorynus salebrosus* (Casey)	X**		
*Stethobaris ovata* (LeConte)	X	X	
**Tribe Baridini Schönherr**			
*Cosmobaris americana* Casey	X		
*Plesiobaris disjuncta* Casey	X***		
**Tribe Madarini Jekel**			
*Madarellus undulatus* (Say)		X	
*Orchidophilus aterrimus* (Waterhouse) ^$^		X	
**Subfamily Ceutorhynchinae Gistel**			
**Tribe Ceutorhynchini Gistel**			
*Amalus scortillum* (Herbst) ^$^	X	X	
*Ceutorhynchus americanus* Buchanan	X	X	
*Ceutorhynchus obstrictus* (Marsham)^$^	X**		
*Ceutorhynchus erysimi* (Fabricius) ^$^	X	X	X
*Ceutorhynchus hamiltoni* Dietz	X	X	X
*Ceutorhynchus neglectus* Blatchley	X		
*Ceutorhynchus omissus* Fall	X	X	
*Ceutorhynchus oregonensis* Dietz		X	
*Ceutorhynchus pallidactylus* (Marsham) ^$^		X	
*Ceutorhynchus semirufus* LeConte	X		
*Ceutorhynchus typhae* (Herbst) ^$^	X	X	
*Glocianus punctiger* (Sahlberg) ^$^	X	X	X
*Hadroplontus litura* (Fabricius) ^$^		X	
*Trichosirocalus horridus* (Panzer) ^$^		X	
**Tribe Cnemogonini Colonnelli**			
*Acanthoscelidius acephalus* (Say)	X	X	X
*Auleutes epilobii* (Paykull)	X	X	X
*Auleutes nebulosus* (LeConte)	X	X	X
*Auleutes tenuipes* (LeConte)	X		
*Cnemogonus lecontei* Dietz	X		
*Perigaster cretura* (Herbst)	X		
*Perigaster liturata* (Dietz)	X	X	X
**Tribe Mononychini LeConte**			
*Mononychus vulpectulus* (Fabricius)	X		
**Tribe Phytobiini Gistel**			
*Parenthis* sp. (undescribed)	X	X	
*Pelenomus fuliginosus* (Dietz)	X	X	
*Pelenomus sulcicollis* (Fabricius)	X**		
*Rhinoncus castor* (Fabricius) ^$^	X	X	X
*Rhinoncus pericarpius* (Linnaeus) ^$^	X	X	X
*Rhinoncus pyrrhopus* Boheman	X	X	X
**Tribe Scleropterini Schultze**			
*Acallodes saltoides* Dietz	X	X	
*Rutidosoma decipiens* (LeConte)			X
**Subfamily Conoderinae Schönherr**			
**Tribe Lechriopini Lacordaire**			
*Acoptus suturalis* LeConte	X	X	
*Lechriops oculata* (Say)	X*	X	X
**Tribe Zygopini Lacordaire**			
*Cylindrocopturus longulus* (LeConte)	X**		
**Subfamily Cossoninae Schönherr**			
**Tribe Cossonini Schönherr**			
*Cossonus americanus* Buchanan	X*	X	
*Cossonus platalea* Say	X	X	
**Tribe Onycholipini Wollaston**			
*Stenoscelis brevis* (Boheman)	X*	X	
**Tribe Rhyncolini Gistel**			
*Carphontus testaceus* Casey	X	X	X
*Himatium errans* LeConte	X*	X	
*Rhyncolus brunneus* Mannerheim	X	X	X
*Rhyncolus macrops* Buchanan		X	
*Phloeophagus apionides* Horn	X*	X	
*Phloeophagus canadensis* Van Dyke	X**		
*Phloeophagus minor* Horn	X**		
**Subfamily Cryptorhynchinae Schönherr**			
**Tribe Cryptorhynchini Schönherr**			
*Cryptorhynchus lapathi* (Linnaeus)	X	X	X
*Eubulus parochus* (Herbst)	X		
*Tyloderma nigrum* Casey	X		
**Subfamily Cyclominae Schönherr**			
**Tribe Listroderini LeConte**			
*Listronotus alternatus* (Dietz)	X	X	X
*Listronotus appendiculatus* (Boheman)	X		
*Listronotus caudatus* (Say)	X		
*Listronotus deceptus* (Blatchley)	X**		
*Listronotus delumbis* (Gyllenhal)	X	X	
*Listronotus dietzi* O’Brien		X	
*Listronotus humilis* (Gyllenhal)	X		
*Listronotus laramiensis* (Angell)	X		
*Listronotus lutulentus* (Boheman)	X**		
*Listronotus maculicollis* (Kirby)	X	X	
*Listronotus oregonensis* (LeConte)	X*	X	
*Listronotus sparsus* (Say)	X	X	X
*Listronotus squamiger* (Say)	X	X	
*Listronotus tuberosus* LeConte	X		
**Subfamily Entiminae Schönherr**			
**Tribe Brachyderini Schönherr**			
*Strophosoma melanogrammum* (Forster) ^$^		X	X
**Tribe Cneorhinini Lacordaire**			
*Philopedon plagiatum* (Schaller) ^$^	X	X	X
**Tribe Geonemini Gistel**			
*Barynotus moerens* (Fabricius) ^$^		X	
*Barynotus obscurus* (Fabricius) ^$^	X	X	X
*Barynotus schoenherri* Zetterstedt^$^	X	X	X
**Tribe Hormorini Horn**			
*Hormorus undulatus* (Uhler)	X	X	X
**Tribe Otiorhynchini Schönherr**			
*Otiorhynchus ligneus* (Olivier) ^$^	X	X	X
*Otiorhynchus ovatus* (Linnaeus) ^$^	X	X	X
*Otiorhynchus raucus* (Fabricius) ^$^		X	
*Otiorhynchus rugifrons* (Gyllenhal) ^$^	X	X	
*Otiorhynchus rugostriatus* (Goeze) ^$^		X	
*Otiorhynchus scaber* (Linnaeus) ^$^		X	
*Otiorhynchus singularis* (Linnaeus) ^$^	X	X	X
*Otiorhynchus sulcatus* (Fabricius) ^$^	X	X	X
**Tribe Peritelini Lacordaire**			
*Nemocestes horni* Van Dyke	X	X	
**Tribe Phyllobiini Schönherr**			
*Phyllobius intrusus* Kôno^$^	X		
*Phyllobius oblongus* (Linnaeus) ^$^	X	X	X
**Tribe Polydrusini Schönherr**			
*Pachyrhinus elegans* (Couper)	X	X	
*Polydrusus cervinus* (Linnaeus) ^$^		X	X
*Polydrusus impressifrons* (Gyllenhal) ^$^	X	X	
*Polydrusus sericeus* (Schaller) ^$^	X	X	X
**Tribe Sciaphilini Sharp**			
*Barypeithes pellucidus* (Beheman) ^$^	X	X	X
*Sciaphilus asperatus* (Bonsdorff) ^$^	X	X	X
**Tribe Sitonini Gistel**			
*Sitona cylindricollis* (Fahraeus) ^$^	X	X	X
*Sitona hispidulus* (Fabricius) ^$^	X	X	X
*Sitona lepidus* Gyllenhal^$^	X	X	X
*Sitona lineelus* (Bonsdorff) ^$^	X	X	X
**Tribe Trachyphloeini Gistel**			
*Trachyphloeus aristatus* (Gyllenhal) ^$^		X	X
*Trachyphloeus bifoveolatus* (Beck) ^$^	X	X	X
*Trachyphloeus spinosus* (Goeze) ^$^		X	
**Tribe Tropiphorini Marseul**			
*Phyxelis rigidus* (Say)	X	X	X
*Tropiporus obtusus* (Bonsdorff) ^$^		X	
*Tropiphorus terricola* (Newman) ^$^	X	X	X
**Subfamily Hyperinae MarseulTribe Hyperini Marseul**			
*Hypera castor* (LeConte)	X	X	X
*Hypera compta* (Say)	X**		
*Hypera meles* (Fabricius) ^$^	X	X	X
*Hypera nigrirostris* (Fabricius) ^$^	X	X	X
*Hypera postica* (Gyllenhal) ^$^	X	X	X
*Hypera zoilus* (Scopoli) ^$^	X	X	X
**Subfamily Lixinae Schönherr**			
**Tribe Cleonini Schönherr**			
*Cleonis pigra* (Scopoli) ^$^	X		
*Scaphomorphus calandroides* (Randall)	X		
**Tribe Lixini Schönherr**			
*Larinus planus* (Fabricius) ^$^		X	
*Lixus rubellus* Randall	X**		
*Rhinocyllus conicus* (Frölich)		X	
**Subfamily Mesoptiliinae Lacordaire**			
**Tribe Magdalidini Lacordaire**			
*Magdalis alutacea* LeConte	X**		
*Magdalis armicollis* Say	X	X	
*Magdalis barbita* (Say)	X*	X	X*
*Magdalis gentilis* LeConte	X	X	
*Magdalis hispoides* LeConte	X**		
*Magdalis piceae* Buchanan		X	
*Magdalis perforata* Horn	X*	X	
*Magdalis salicis* Horn		X	
**Subfamily Molytinae Schönherr**			
**Tribe Conotrachelini Jekel**			
*Conotrachelus anaglypticus* (Say)	X		
*Conotrachelus juglandis* LeConte	X**		
*Conotrachelus nenuphar* (Herbst)	X	X	
*Conotrachelus posticatus* Boheman	X*	X	
**Tribe Hylobiini Kirby**			
*Hylobius congener* Dalla Torre et al.	X	X	X
*Hylobius pales* (Herbst)	X	X	
*Hylobius pinicola* (Couper)	X	X	
*Hylobius transversovittatus* (Goeze) ^$^		X	
*Hylobius warreni* Wood	X	X	
**Tribe Molytini Schönherr**			
*Sthereus ptinoides* (Germar)	X*	X	
**Tribe Pissodini Gistel**			
*Pissodes affinis* Randall	X	X	
*Pissodes fiskei* Hopkins	X	X	X
*Pissodes nemorensis* Germar	X	X	X
*Pissodes rotundatus* LeConte	X	X	
*Pissodes similis* Hopkins	X	X	
*Pissodes striatulus* (Fabricius)	X	X	X
*Pissodes strobi* (Peck)	X	X	X
**Subfamily Scolytinae Latreille**			
**Tribe Corythylini LeConte**			
*Gnathotrichus materarius* (Fitch)	X	X	
*Conophthorus coniperda* (Schwartz)	X	X	
*Conophthorus resinosae* Hopkins	X	X	
*Corthylus columbianus* Hopkins		X	
*Monarthrum mali* (Fitch)	X	X	
*Pityophthorus angustus* Blackman	X	X	
*Pityophthorus balsameus* Blackman	X	X	
*Pityophthorus biovalis* Blackman	X*	X	
*Pityophthorus briscoei* Blackman	X	X	
*Pityophthorus carinatus* Bright	X	X	
*Pityophthorus cariniceps* LeConte	X	X	
*Pityophthorus concavus* Blackman	X	X	
*Pityophthorus consimilis* LeConte		X	
*Pityophthorus dentifrons* Blackman	X	X	X
*Pityophthorus intextus* Swaine	X	X	
*Pityophthorus murrayanae* Blackman	X		
*Pityophthorus nitidus* Swaine	X	X	
*Pityophthorus opaculus* LeConte	X	X	
*Pityophthorus puberulus* (LeConte)	X	X	
*Pityophthorus pulchelus* Eichhoff	X	X	
*Pityophthorus pulicarius* (Zimmerman)	X	X	
*Pityophthorus ramiperda* Swaine		X	
*Pseudopityophthorus minutissimus* (Zimmermann)	X*	X	
**Tribe Cryphalini Lindermann**			
*Trypophloeus populi* Hopkins	X		
*Trypophloeus striatulus* (Mannerheim)		X	
*Cryphalus ruficollis* Hopkins	X	X	
**Tribe Crypturgini LeConte**			
*Crypturgus borealis* Swaine	X	X	X
*Crypturgus pusillus* (Gyllenhal) ^$^	X	X	X
**Tribe Dryocoetini Lindemann**			
*Dryocoetes affaber* (Mannerheim)	X	X	X
*Dryocoetes autographus* (Ratzeburg)	X	X	X
*Dryocoetes betulae* Hopkins	X	X	
*Dryocoetes caryi* Hopkins	X**	X**	
*Lymantor decipiens* (LeConte)		X	
**Tribe Hylastini LeConte**			
*Hylastes porculus* Erichson	X	X	X
*Hylastes opacus* Erichson^$^	X**		
*Hylurgops rugipennis pinifex* (Fitch)	X	X	X
*Scierus annectans* LeConte	X	X	X
**Tribe Hylesinini Erichson**			
*Hylastinus obscurus* (Marsham) ^$^		X	
*Hylesinus aculeatus* (Say)	X*	X	
**Tribe Hylurgini Gistel**			
*Dendroctonus punctatus* LeConte	X		
*Dendroctonus rufipennis* (Kirby)	X	X	X
*Dendroctonus simplex* LeConte	X	X	X
*Dendroctonus valens* LeConte	X	X	
*Hylurgopinus rufipes* (Eichhoff)	X	X	
*Xylechinus americanus* Blackman	X*	X	
**Tribe Ipini Bedel**			
*Ips borealis* Swaine	X	X	X
*Ips grandicollis* (Eichhoff)		X	
*Ips perroti* Swaine	X		
*Ips perturbatus* (Eichhoff)	X		
*Ips pini* (Say)	X	X	X*
*Orthotomicus caelatus* (Eichhoff)	X	X	X
*Orthotomicus latidens* (LeConte)	X**	X**	
*Pityogenes hopkinsi* Swaine	X	X	
*Pityogenes plagiatus* (LeConte)	X**		
*Pityokteines sparsus* (LeConte)	X	X	X
**Tribe Phloeosinini Nüsslin**			
*Phloeosinus canadensis* Swaine	X		
*Phloeosinus pini* Swaine		X	
**Tribe Phloeotribini Chapius**			
*Phloeotribus liminaris* (Harris)	X	X	
*Phloeotribus piceae* Swaine	X	X	
**Tribe Polygraphini Chapuis**			
*Polygraphus rufipennis* (Kirby)	X	X	X
*Carphoborus carri* Swaine	X		
*Carphoborus dunni* Swaine	X		
**Tribe Scolytini Latreille**			
*Scolytus multistriatus* (Marsham) ^$^	X	X	
*Scolytus piceae* (Swaine)	X	X	
*Scolytus rugulosus* (Muller) ^$^	X	X	X
**Tribe Xyloborini LeConte**			
*Anisandrus dispar* (Fabricius) ^$^	X*	X	X
*Anisandrus obesus* LeConte	X*	X	
*Anisandrus sayi* (Hopkins)	X*	X	
*Xyleborinus attenuatus* (Blanford)			X
*Xyleborinus saxesenii* (Ratzeburg) ^$^	X*	X	
*Xyleborus atratus* Eichhoff^$^		X	
*Xylosandrus germanus* (Balndford) ^$^		X	
**Tribe Xyloterini LeConte**			
*Trypodendron betulae* Swaine	X	X	X
*Trypodendrum domesticum* (Linnaeus) ^$^			X
*Trypodendron lineatum* (Olivier)	X	X	X
*Trypodendron retusum* (LeConte)	X	X	X
*Trypodendron rufitarsis* (Kirby)	X	X	
*Xyloterinus politus* (Say) ^$^	X	X	X
**Total number of species**	**269**	**254**	**100**

**Notes:** *New to province, **New to Maritime provinces, ***New to Canada, ^$^Adventive species.

### Species accounts

All records below are species newly recorded for New Brunswick, Nova Scotia, or Prince Edward Island, Canada, unless noted otherwise (additional records). Species followed by ** are newly recorded from the Maritime provinces; species followed by *** are newly recorded for Canada.

### Family Antribidae Billberg, 1820

**Subfamily Anthribinae Billberg, 1820**

**Tribe Tropiderini Lacordaire, 1865**

#### Eurymycter latifascia

Pierce, 1930

http://species-id.net/wiki/Eurymycter_latifascia

Map 1

##### Material examined.

**New Brunswick, Charlotte Co.**, 10 km NW of New River Beach, 45.2110°N, 66.6170°W, 31.V–15.VI.2010, R. Webster & C. MacKay, old growth eastern white cedar forest, Lindgren funnel trap (1, RWC).

**Map 1.**
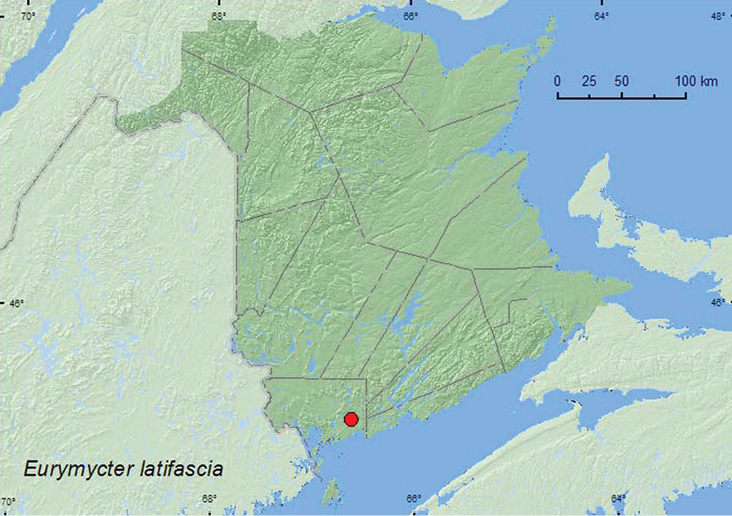
Collection localities in New Brunswick, Canada of *Eurymycter latifascia*.

##### Collection and habitat data.

*Eurymycter* spp. feed on *Daldinia* and *Hypoxylon* spp. fungi (Xylariaceae) (Valentine 1999). This species was captured in a Lindgren funnel trap deployed in an old-growth eastern white cedar (*Thuja occidentalis* L.) forest in June.

##### Distribution in Canada and Alaska.

ON,QC, **NB,** NS(McNamara 1991a; Bright 1993).

### Tribe Zygaenodini Lacordaire, 1865

#### Ormiscus saltator

LeConte, 1876**

http://species-id.net/wiki/Ormiscus_saltator

Map 2

##### Material examined.

**New Brunswick, York Co.**, 15 km W of Tracy off Rt. 645, 45.6848°N, 66.8821°W, 13–27.VII.2010, R. Webster & C. MacKay, old red pine forest, Lindgren funnel traps (2, RWC).

**Map 2.**
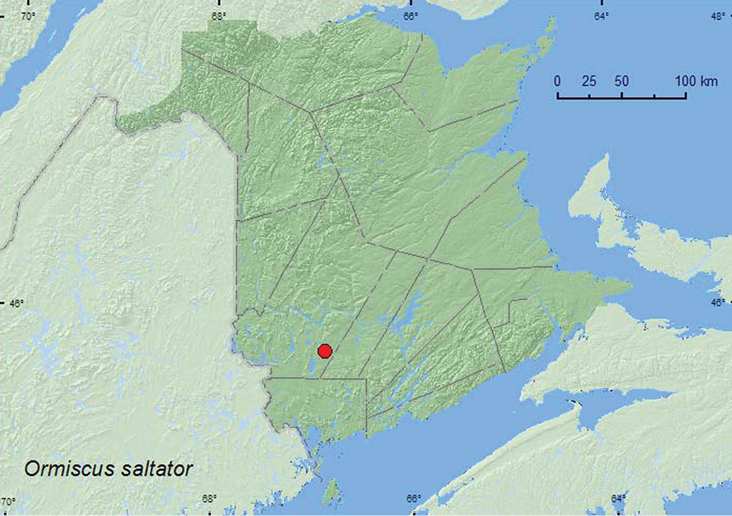
Collection localities in New Brunswick, Canada of *Ormiscus saltator*.

##### Collection and habitat data.

This species was captured during July in Lindgren funnel traps in an old red pine (*Pinus resinosa* Ait.) forest.

##### Distribution in Canada and Alaska.

ON, QC, **NB** (McNamara 1991a).

### Subfamily Choraginae Kirby, 1819

**Tribe Choragini Kirby, 1819**

#### Choragus sayi

LeConte, 1876**

http://species-id.net/wiki/Choragus_sayi

Map 3

##### Material examined.

**New Brunswick, Charlotte Co.**, 10 km NW of New River Beach, 45.2110°N, 66.6170°W, 16–26.VII.2010, R. Webster & C. MacKay, old growth eastern white cedar forest, Lindgren funnel trap (1, RWC). **Queens Co.**, Grand Lake Meadows P.N.A. (Protected Natural Area), 45.8227°N, 66.1209°W, 26.VII–7.VIII.2010, R. Webster, old silver maple forest with green ash and seasonally flooded marsh, Lindgren funnel trap (1, RWC); same locality data and forest type, M. Roy & V. Webster, 19.VII–5.VIII.2011, Lindgren funnel traps (2, AFC, NBM); Cranberry Lake P.N.A., 46.1125°N, 65.6075°W, 29.VI–7.VII.2011, 7–13.VII.2011, 20.VII–4.VIII.2011, 4–18.VIII.2011, M. Roy & V. Webster, old red oak forest, Lindgren funnel traps (7, AFC, NBM, RWC); same locality data and forest type, C. Hughes & R. P. Webster, 18–31.VIII.2011, Lindgren funnel traps (2, RWC).

**Map 3.**
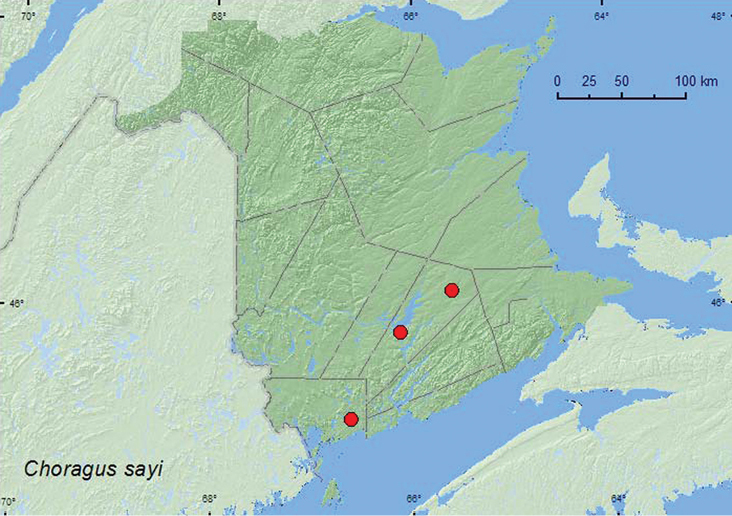
Collection localities in New Brunswick, Canada of *Choragus sayi*.

##### Collection and habitat data.

This species was captured during July and August in Lindgren funnel traps in an old-growth eastern white cedar forest, an old red oak (*Quercus rubra* L.) forest, and an old silver maple (*Acer saccharinum* L.) forest.

##### Distribution in Canada and Alaska.

QC**, NB** (Valentine 1998).

### Family Brentidae Billberg, 1820

**Subfamily Apioninae Schönherr, 1823**

**Tribe Apionini Schönherr, 1823**

#### Coelocephalapion emaciipes

(Fall, 1898)

http://species-id.net/wiki/Coelocephalapion_emaciipes

Map 4

##### Material examined.

**New Brunswick, York Co.**, 15 km W of Tracy off Rt. 645, 45.6848°N, 66.8821°W, 30.V-8.VI.2011, R. Roy & V. Webster, old red pine forest, Lindgren funnel trap (1, RWC).

**Map 4.**
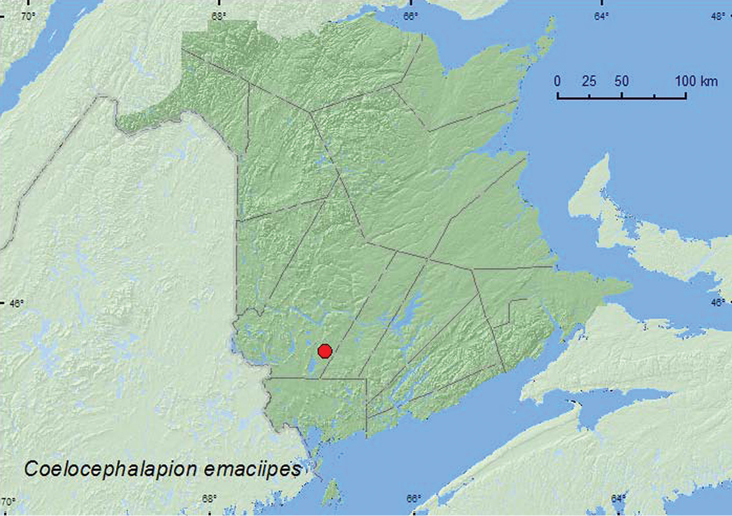
Collection localities in New Brunswick, Canada of *Coelocephalapion emaciipes*.

##### Collection and habitat data.

This species is associated with tick-trefoil (*Desmodium* spp. ) (Bright 1993). The specimen from New Brunswick was captured between late May and early June in a Lindgren funnel trap in an old red pine forest.

##### Distribution in Canada and Alaska.

ON, **NB**, NS, PE (McNamara 1991b; Majka et al. 2007a; Majka 2010a). This species was newly recorded from Nova Scotia and the Maritime provinces by Majka et al. (2007a).

#### Neapion frosti

(Kissinger, 1968)

http://species-id.net/wiki/Neapion_frosti

Map 5

##### Material examined.

**New Brunswick, York Co.**, 15 km W of Tracy off Rt. 645, 45.6837°N, 66.8809°W, 16.VI.2007, 28.VI.2007, R. P. Webster, old red pine forest, on flowers of *Viburnum nudum* (12, NBM, RWC); Charters Settlement, 45.8430°N, 66.7275°W, 17.VI.2007, R. P. Webster, regenerating mixed forest, sweeping flowers of *Viburnum nudum* (1, RWC).

**Map 5.**
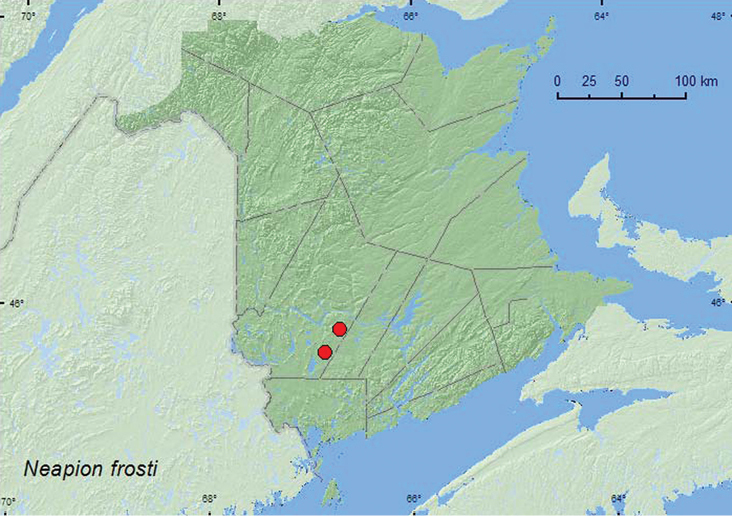
Collection localities in New Brunswick, Canada of *Neapion frosti*.

##### Collection and habitat data.

*Neapion* sp. are associated with *Viburnum* (Anderson and Kissinger 2002). The New Brunswick specimens of *Neapion frosti* were collected in June from flowers of wild raisin (*Viburnum nudum* L.) in an old red pine forest and in a regenerating mixed forest.

##### Distribution in Canada and Alaska.

ON, QC, **NB**, NS (McNamara 1991b).

#### Podapion gallicola

Riley, 1883**

http://species-id.net/wiki/Podapion_gallicola

Map 6

##### Material examined.

**New Brunswick, Sunbury Co.**, Otter Brook Rd., off Little Lake Rd. emerged. 24.III.1969, reared from red pine, (no collector given) 68–2-1869–01 (1, AFC). **York Co.**, 15 km W of Tracy off Rt. 645, 45.6848°N, 66.8821°W, 14–20.VII.2009, 20–29.VII.2009, 4–11.VIII.2009, R. Webster & M.-A. Giguère, old red pine forest, Lindgren funnel traps (3, RWC); same data, but 13–27.VII.2010, 27.VII–10.VIII.2010, R. Webster & C. MacKay, old red pine forest, Lindgren funnel traps (2, RWC); 14 km WSW of Tracy, S of Rt. 645, 45.6741°N, 66.8661°W, 13–27.VII.2010, R. Webster & C. MacKay, old mixed forest with red and white spruce, red and white pine, balsam fir, eastern white cedar, red maple, and *Populus* sp., Lindgren funnel trap (1, RWC).

**Map 6.**
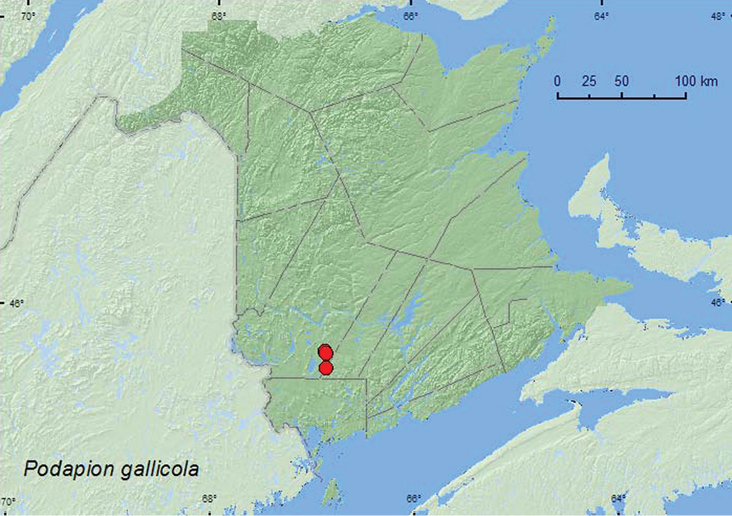
Collection localities in New Brunswick, Canada of *Podapion gallicola*.

##### Collection and habitat data.

This species is associated with *Pinus* spp., and larvae occur in galls on twigs (Anderson and Kissinger 2002). In New Brunswick, adults were captured during July and August in Lindgren funnel traps deployed in an old red pine forest and an old mixed forest with red pine. One specimen was reared from red pine.

##### Distribution in Canada and Alaska.

ON, QC, **NB** (McNamara 1991b).

#### Trichapion porcatum

(Boheman, 1839)**

http://species-id.net/wiki/Trichapion_porcatum

Map 7

##### Material examined.

**New Brunswick, Queens Co.**, near Stony Point off Rt. 690, 46.0364°N, 66.0383°W, 12.VII.2006, R. P. Webster, on foliage of *Robinia pseudoacacia* L. (2, RWC).

**Map 7.**
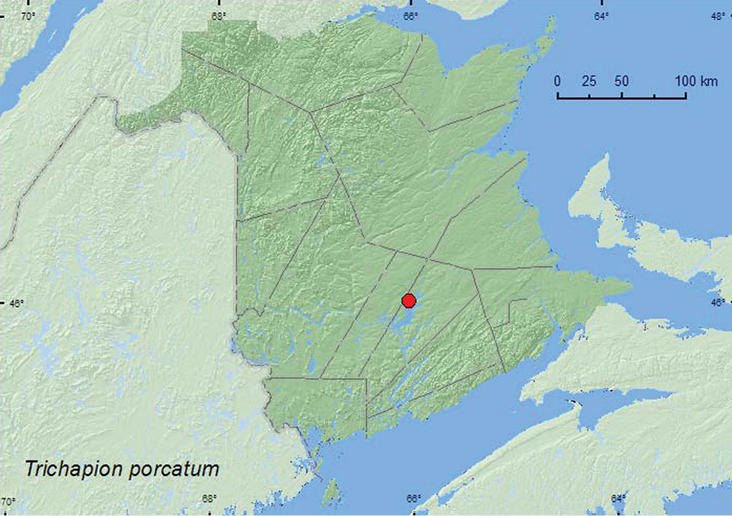
Collection localities in New Brunswick, Canada of *Trichapion porcatum*.

##### Collection and habitat data.

Two individuals were beaten from foliage of black locust (*Robinia pseudoacacia* L.) during mid July.

##### Distribution in Canada and Alaska.

ON, **NB** (McNamara 1991b).

### Family Dryophthoridae Schönherr, 1825

**Subfamily Dryophthorinae Schönherr, 1825**

**Tribe Rhynchophorini Schönherr, 1833**

#### Sitophilus oryzae

(Linnaeus, 1758)

http://species-id.net/wiki/Sitophilus_oryzae

Map 8

##### Material examined.

**New Brunswick, Westmorland Co.**, Moncton, 21.IV.1945, 24.IV.1945, R. S. Forbes (3, AFC).

**Map 8.**
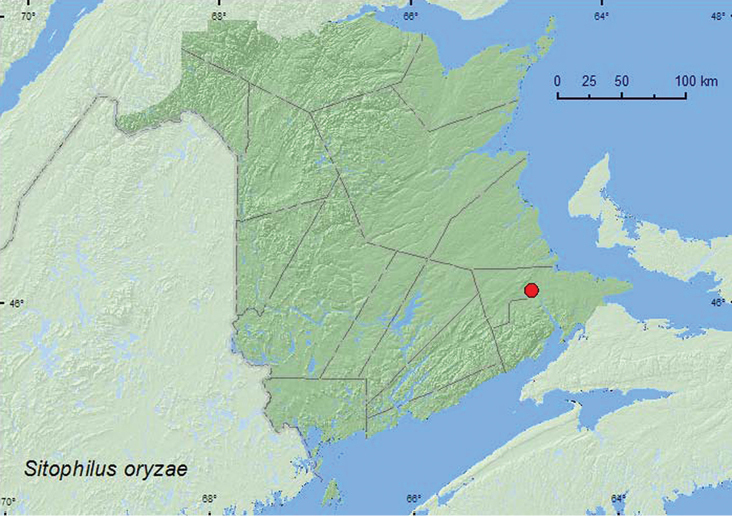
Collection localities in New Brunswick, Canada of *Sitophilus oryzae*.

##### Collection and habitat data.

This introduced cosmopolitan species is a pest of stored grain products worldwide (Anderson 2002).No bionomic data are associated with the specimens of this species from New Brunswick.

##### Distribution in Canada and Alaska. 

BC, ON, QC, **NB,** NS, PE, NF (McNamara 1991c; McCorquodale et al. 2005; Majka et al. 2007c).

### Tribe Sphenophorini Lacordaire, 1865

#### Sphenophorus parvulus

Gyllenhal, 1838

http://species-id.net/wiki/Sphenophorus_parvulus

Map 9

##### Material examined.

**New Brunswick, York Co.**, Charters Settlement, 45.8395°N, 66.7391°W, 12.VII.2005, R. P. Webster, roadside, on pavement (1, RWC).

**Map 9.**
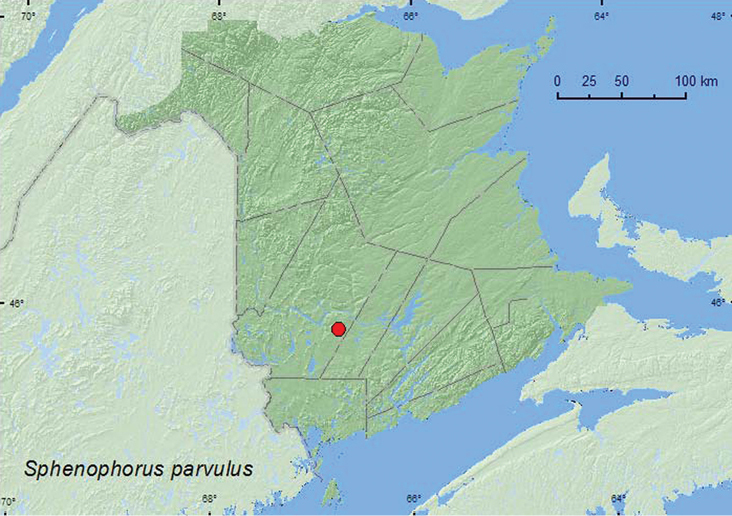
Collection localities in New Brunswick, Canada of *Sphenophorus parvulus*.

##### Collection and habitat data.

*Sphenophorus parvulus* (the bluegrass billbug) feeds on Kentucky bluegrass, *Poa pratensis* L. and other grasses, and is an important turf pest in the United States (Vaurie 1951; Tashiro and Personius 1970; Kindler and Kinbacher 1975; Kindler and Spomer 1986). The specimen from New Brunswick was found on the side of a residential street during July.

##### Distribution in Canada and Alaska.

ON, QC, **NB**, NS, (McNamara 1991c; Majka et al. 2007c).

#### Sphenophorus zeae

Walsh, 1867

http://species-id.net/wiki/Sphenophorus_zeae

Map 10

##### Material examined.

**New Brunswick, York Co.**, Charters Settlement, 45.8404°N, 66.7360°W, 27.V.2008, R. P. Webster, medium sized brook partially shaded by alders, among *Carex* (1, RWC).

**Map 10.**
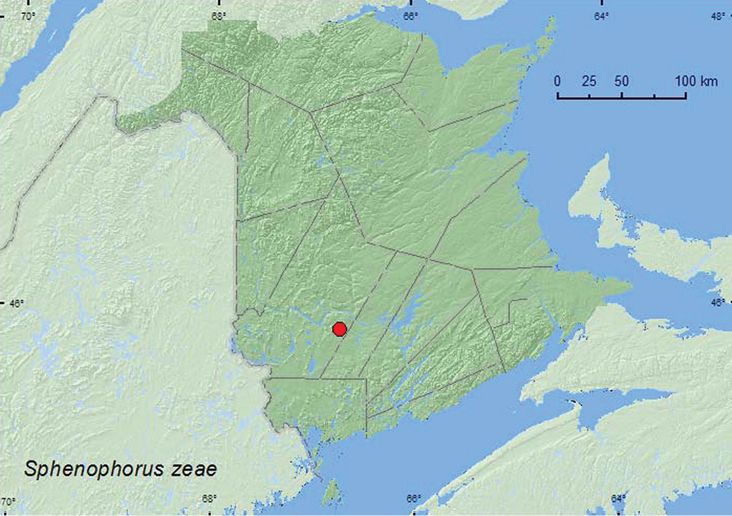
Collection localities in New Brunswick, Canada of *Sphenophorus zeae*.

##### Collection and habitat data.

Majka et al. (2007b) reported this species from coastal salt-spray barrens in Nova Scotia. This species feeds on grasses (Poaceae) such as *Poa pratensis* L., *Phleum pratense* L., and *Zea mays* (L.) (Vaurie 1951). The specimen from New Brunswick was found in a *Carex* hummock near a brook during May.

##### Distribution in Canada and Alaska.

ON, QC, **NB**, NS, PE(McNamara 1991c; Majka et al. 2007c).

### Family Brachyceridae Billberg, 1820

**Subfamily Erirhininae Schönherr, 1825**

**Tribe Erirhinini Schönherr, 1825**

#### Notiodes ovalis

(LeConte, 1876)**

http://species-id.net/wiki/Notiodes_ovalis

Map 11

##### Material examined.

**New Brunswick, York Co.**, Mazerolle Settlement, 45.8765°N, 66.8260°W, 8.VI.2008, R. P. Webster, beaver meadow, sweeping vegetation along brook margin (3, RWC).

**Map 11.**
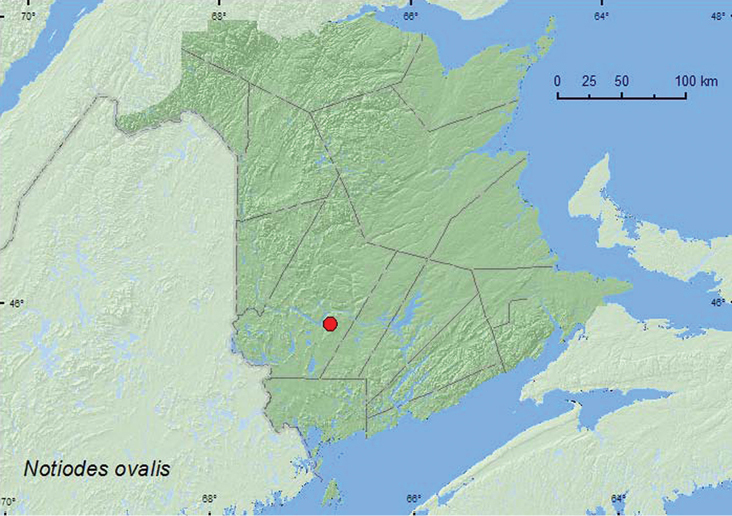
Collection localities in New Brunswick, Canada of *Notiodes ovalis*.

##### Collection and habitat data.

The New Brunswick specimens of *Notiodes ovalis* were swept from vegetation along a brook margin in a beaver (*Castor canadensis* Kuhl.) meadow during June.

##### Distribution in Canada and Alaska.

AB, ON, QC, **NB** (McNamara 1991c).

#### Onychylis nigrirostris

(Boheman, 1843)

http://species-id.net/wiki/Onychylis_nigrirostris

Map 12

##### Material examined.

**New Brunswick, Queens Co.**, Grand Lake Meadows P.N.A., 45.8227°N, 66.1209°W, 17–30.VIII.2011, C. Hughes & R. P. Webster, old silver maple forest with green ash and seasonally flooded marsh, Lindgren funnel trap (1, RWC).

**Map 12.**
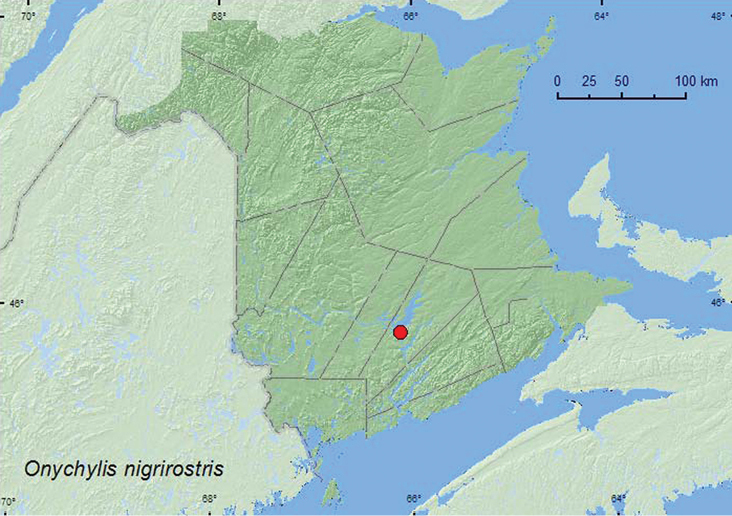
Collection localities in New Brunswick, Canada of *Onychylis nigrirostris*

##### Collection and habitat data.

*Onychylis* spp. are associated with pickerelweed (*Pontederia cordata* L.) and pond lilies (*Nuphar* species) (Anderson 1993). The specimen from New Brunswick was captured during August in a Lindgren funnel trap in an old silver maple swamp near a seasonally flooded marsh.

##### Distribution in Canada and Alaska.

ON, QC, **NB**, NS(McNamara 1991c; Majka et al. 2007c).

### Tribe Tanysphyrini Gistel, 1848

#### Tanysphrus lemnae

(Fabricius, 1792)

http://species-id.net/wiki/Tanysphrus_lemnae

Map 13

##### Material examined.

**New Brunswick, Queens Co.**, just W of Jemseg at “Trout Creek”, 45.8231°N, 66.1245°W, 11.IV.2006, R. P. Webster, silver maple swamp, sifting litter from silver maple with multiple trunks (1, RWC); Grand Lake Meadows P.N.A., 45.8227°N, 66.1209°W, 29.VI–12.VII.2010, R. Webster, C. MacKay, M. Laity & R. Johns, old silver maple forest with green ash and seasonally flooded marsh, Lindgren funnel traps (1, AFC). **York Co.** Charters Settlement, 45.8456°N, 66.7267°W, 1.V.2010, 5.V.2010, R. P. Webster, beaver pond, on *Lemna* sp. on pond margin (6, RWC).

**Map 13.**
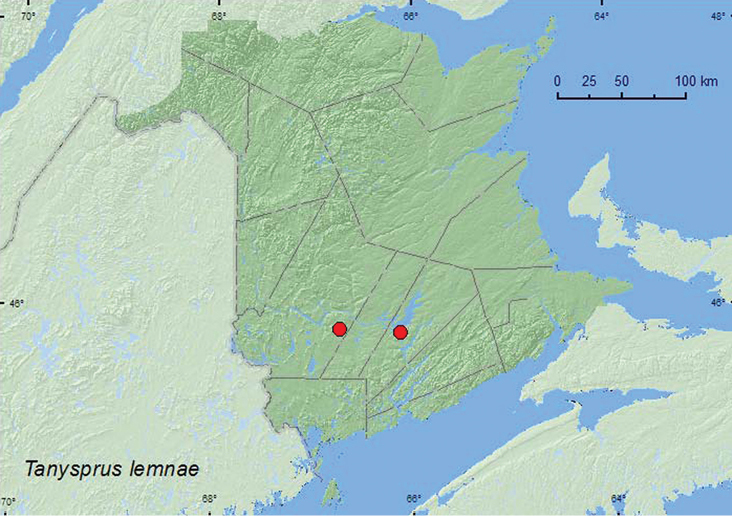
Collection localities in New Brunswick, Canada of *Tanysphrus lemnae*.

##### Collection and habitat data.

This species is associated with *Lemna* spp. (Lemnaceae) (Anderson 2002). Most of the specimens from New Brunswick were found on *Lemna* sp. floating on water near the margin of a beaver pond during May. One individual was sifted from litter in the crotch of a silver maple with multiple trunks during mid April (probably an overwintering site) and another was captured during July in a Lindgren funnel trap in an old silver maple swamp.

##### Distribution in Canada and Alaska.

BC, AB, SK, MB, ON, QC, **NB**, NS (McNamara 1991c; Majka et al. 2007c).

### Family Curculionidae Latreille, 1802

**Subfamily Curculioninae Latreille, 1802**

**Tribe Anthonomini Thomson, 1859**

#### Anthonomus haematopus

Boheman, 1843

http://species-id.net/wiki/Anthonomus_haematopus

Map 14

##### Material examined.

**New Brunswick, Charlotte Co.**, near Clark Ridge, 45.3040°N, 67.4252°W, 27.V.2007, R. P. Webster, old field, on Salix foliage (1, RWC). **Gloucester Co.**, Bass River, 20.III.1970, (no collector given) reared from willow (1, AFC). **Restigouche Co.**, Jacquet River Gorge P.N.A., 47.8204°N, 66.0833°W, 14.VI.2009, R. P. Webster, river margin, beating foliage (1, RWC). **York Co.** Charters Settlement, 45.8430°N, 66.7275°W, 17.VI.2007, R. P. Webster, regenerating mixed forest, sweeping foliage in brushy opening (1, RWC).

**Map 14.**
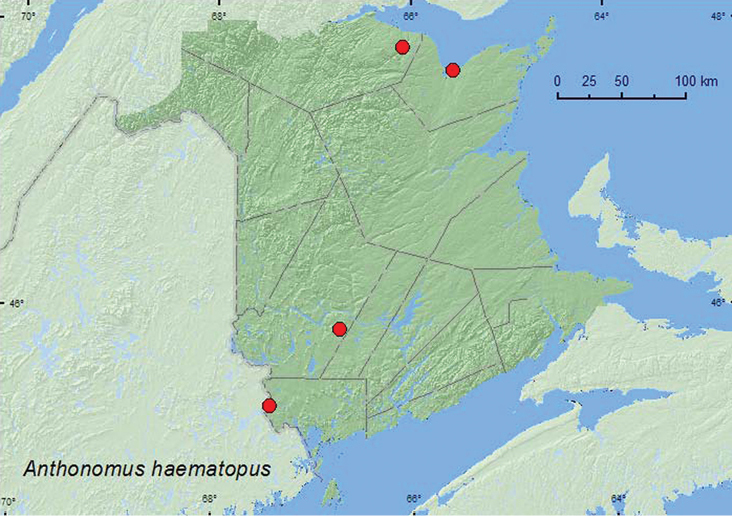
Collection localities in New Brunswick, Canada of *Anthonomus haematopus*.

##### Collection and habitat data.

*Anthonomus haematopus*is associated with galls on *Salix* spp. generated by sawflies (Ahmad and Burke 1972). Adults from New Brunswick were collected in an old field, along a river margin, and in a brushy opening in a regenerating mixed forest. One specimen was on *Salix* foliage, another was reared from *Salix*. Adults were collected during May and June.

##### Distribution in Canada and Alaska.

BC, AB, SK, MB, ON, QC, **NB**, NS (McNamara 1991c).

#### Anthonomus subfasciatus

LeConte, 1876

http://species-id.net/wiki/Anthonomus_subfasciatus

Map 15

##### Material examined.

**New Brunswick, Sunbury Co.**, Lakeville Corner, 45.9008°N, 66.2414°W, 12.VII.2006, R. P. Webster, silver maple swamp on ridge with red maple and red oak, on flowers of *Spiraea alba* (12, NBM, RWC); 9.5 km NE jct. Rt. 101 & 645, 45.7586°N, 66.6755°W, 29.VII.2007, R. P. Webster, old field with open sandy areas, sweeping (1, AFC). **York Co.**, Rt. 645 at Beaver Brook, 45.6860°N, 66.8668°W, 8.VII.2008, 13.VIII.2008, R. P. Webster, sedge marsh, on flowers of *Spiraea alba* (2, RWC); 15 km W of Tracy off Rt. 645, 45.6848°N, 66.8821°W, 4–16.VI.2010, R. Webster & C. MacKay, coll., old red pine forest, Lindgren funnel trap (1, AFC).

**Map 15.**
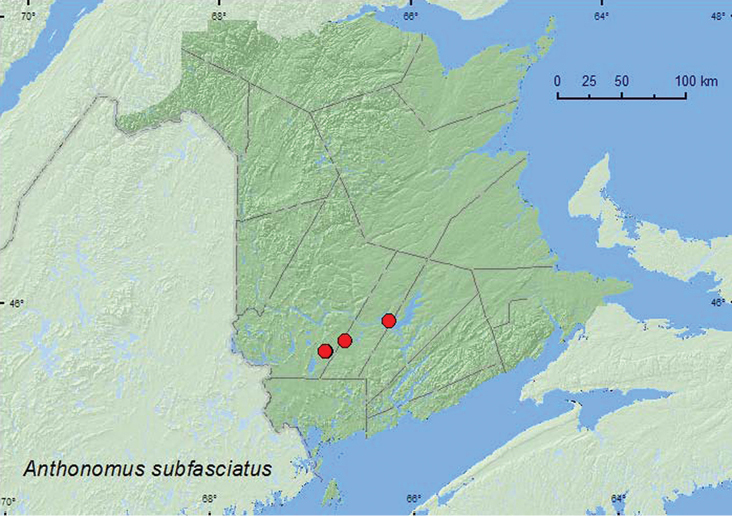
Collection localities in New Brunswick, Canada of *Anthonomus subfasciatus*.

##### Collection and habitat data.

This species has been collected from *Viburnum dentatum* L. (Ahmad and Burke 1972). In New Brunswick, this species was common on flowers of meadow-sweet (*Spiraea alba* Du Roi) at two localities. Adults were collected during July and August.

##### Distribution in Canada and Alaska.

ON, QC, **NB**, NS (McNamara 1991c; Majka et al. 2007c).

### Tribe Curculionini Latreille, 1802

#### Curculio obtusus

(Blanchard, 1884)**

http://species-id.net/wiki/Curculio_obtusus

Map 16

##### Material examined.

**New Brunswick, Carleton Co.**, Bellville, Meduxnekeag Valley Nature Preserve, 46.1890°N, 67.6766°W, 8.VI.2005, 4.VII.2005, R. Webster & M.-A. Giguère, floodplain forest, beating foliage of *Corylus cornuta* (2, RWC); same locality, but 46.1931°N, 67.6825°W, 25.VI.2007, R. P. Webster, floodplain forest, beating foliage of *Corylus cornuta* (2, RWC). **Queens Co.**, Cranberry Lake P.N.A., 46.1125°N, 65.6075°W, 15–21.VII.2009, R. Webster & M.-A. Giguère, old red oak forest, Lindgren funnel trap (1, RWC); same locality data and forest type but 18.VI.2009, R. Webster & M.-A. Giguère, sweeping foliage (1, AFC); same locality data and forest type 29.VI-7.VII.2011, 7–13.VII.2011, 20.VII-4.VIII.2011, M. Roy & V. Webster, Lindgren funnel traps (3, AFC, NBM, RWC). **York Co.**, Canterbury, near “Browns Mountain Fen”, 45.8978°N, 67.6273°W, 3.VII.2005, R. Webster & M.-A. Giguère, mixed forest, on foliage of *Corylus cornuta* (1, RWC).

**Map 16.**
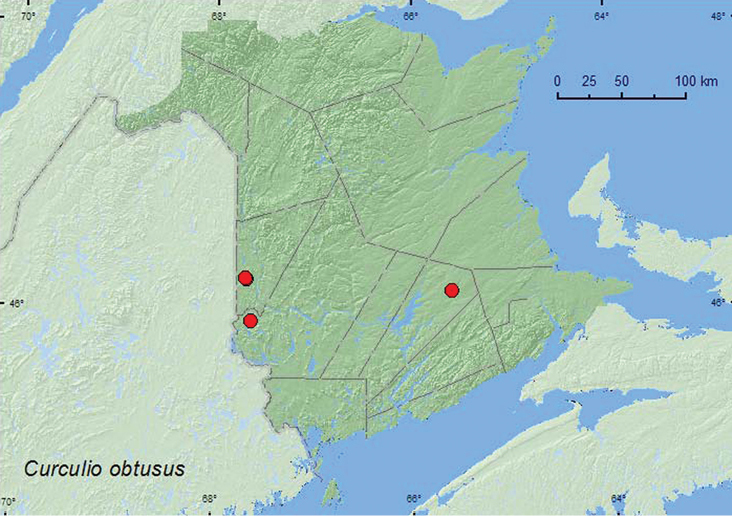
Collection localities in New Brunswick, Canada of *Curculio obtusus*.

##### Collection and habitat data.

Most adults from New Brunswick were collected by beating foliage of beaked hazelnut (*Corylus cornuta* Marsh.) or sweeping foliage near this shrub. Some adults were collected in Lindgren funnel traps in an old red oak forest with abundant *Curculio cornuta* in the understory. Adults were collected during June, July, and August.

##### Distribution in Canada and Alaska.

MB, ON, QC, **NB** (McNamara 1991c).

### Tribe Ellescini Thomson, 1859

#### Ellescus ephippiatus

(Say, 1831)**

http://species-id.net/wiki/Ellescus_ephippiatus

Map 17

##### Material examined.

**New Brunswick, Carleton Co.**, Medunxnekeag Valley Nature Preserve, 46.1878°N, 67.6705°W, 18.VIII.2008, R. P. Webster, hardwood forest, sweeping (1, RWC). **Queens Co.**, Cranberry Lake P.N.A., 46.1125°N, 65.6075°W, 3–13.V.2011, 13–25.V.2011, M. Roy & V. Webster, old red oak forest, Lindgren funnel trap in forest canopy (trap in a big toothed aspen) (15, AFC, NBM, RWC). **York Co.**, Fredericton, 12.V.1921, 19.V.1921, 20.V.1921, R.P.G. (16, AFC).

**Map 17.**
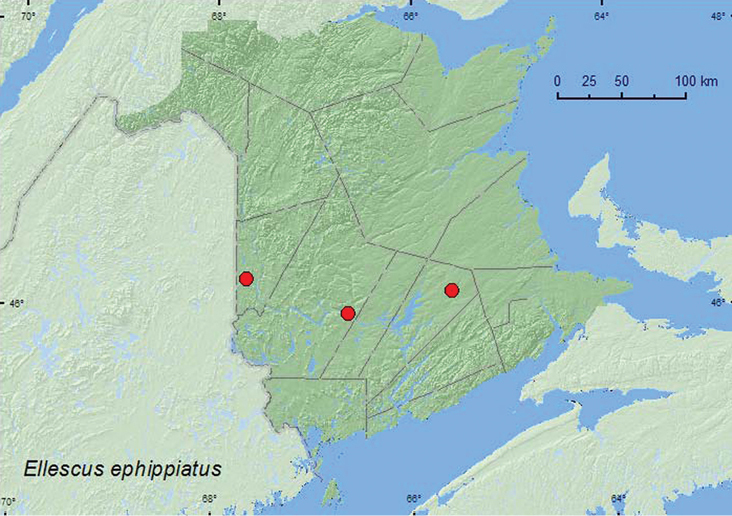
Collection localities in New Brunswick, Canada of *Ellescus ephippiatus*.

##### Collection and habitat data.

Species in this genus are associated with *Salix* and *Populus* spp. (Anderson 2002). The only adult from New Brunswick with bionomic data was swept from foliage in a hardwood forest. Other individuals were captured in Lindgren funnel traps in an old red oak forest deployed in the canopy of a large-toothed aspen (*Populus grandidentata* Michx.) Adults were collected during May and August.

##### Distribution in Canada and Alaska.

MB, ON, QC, **NB** (McNamara 1991c).

#### Dorytomus frostii

Blatchley, 1916**

http://species-id.net/wiki/Dorytomus_frostii

Map 18

##### Material examined.

**New Brunswick, Sunbury Co.**, Acadia Station, emerged 5.V.1958, 6.V.1958, 7.V.1958, 9.V.1958, 12.V.1958 (no collector given), reared from *Populus tremuloides*, F.I.S., 58–0045–01 (2, AFC).

**Map 18.**
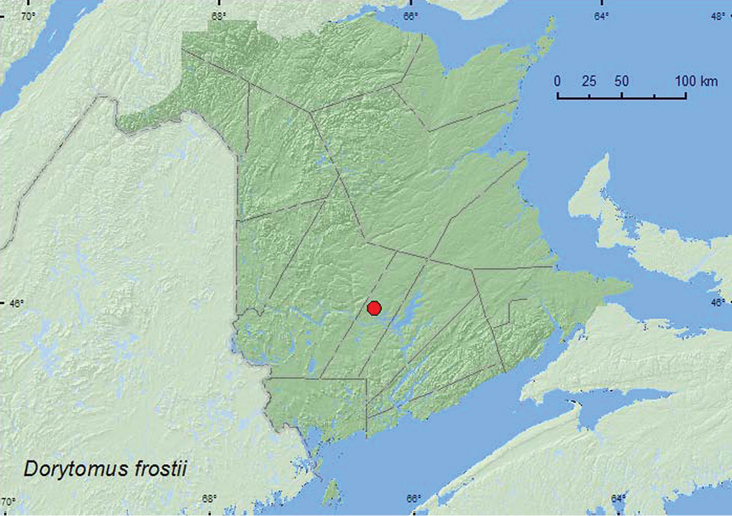
Collection localities in New Brunswick, Canada of *Dorytomus frostii*.

##### Collection and habitat data.

O’Brien (1970) reported trembling aspen, *Populus tremuloides* Michx. as the host of this species. Specimens of this species were reared from *Populus tremuloides* in New Brunswick.

##### Distribution in Canada and Alaska.

YK, NT, BC, AB, SK, MB, ON, QC, **NB** (McNamara 1991c).

#### Dorytomus laticollis

LeConte, 1876

http://species-id.net/wiki/Dorytomus_laticollis

Map 19

##### Material examined.

**New Brunswick, Sunbury Co.**, Acadia Station, emerged 5.V.1958, 6.V.1958, 7.V.1958, 9.V.1958, 12.V.1958, (no collector given) reared from *Populus tremuloides*, F.I.S., 58–0045–01 (48, AFC). **York Co.**, Durham (Bridge), 22.VII.1958, G. W. Barter, ex. *Populus tremuloides* (1, AFC); Kingsley, 14.VIII.1964, Titus, ex. willow (hand picked) F.I.S. 64–1568–04 (1, AFC).

**Map 19.**
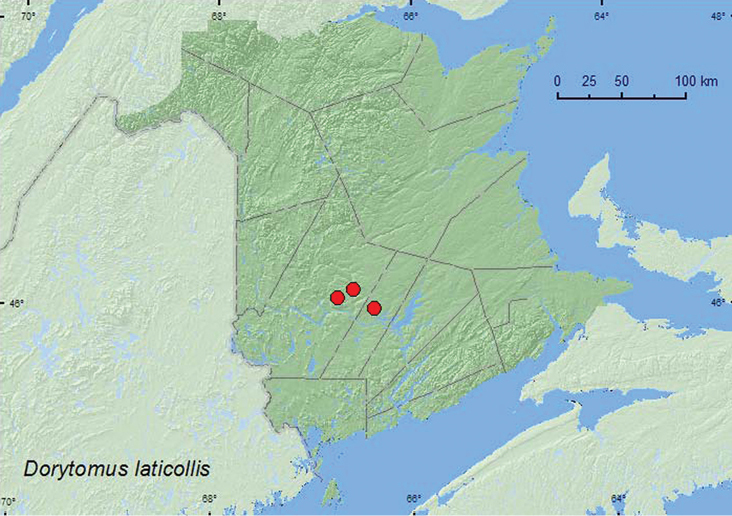
Collection localities in New Brunswick, Canada of *Dorytomus laticollis*.

##### Collection and habitat data.

O’Brien (1970) reported trembling aspen as the host of this species. A large series of this species from New Brunswick was reared from *Populus tremuloides*. Emergence dates were during May and July.

##### Distribution in Canada and Alaska.

AK, BC, AB, SK, MB, ON, QC, **NB**, NS (McNamara 1991c; Majka et al. 2007c).

#### Dorytomus luridus

Mannerheim, 1853

http://species-id.net/wiki/Dorytomus_luridus

Map 20

##### Material examined.

**New Brunswick, Queens Co.**, Cranberry Lake P.N.A., 46.1125°N, 65.6075°W, 29.VI-7.VII.2011, M. Roy & V. Webster, old red oak forest, Lindgren funnel trap in forest canopy (1, RWC).

**Map 20.**
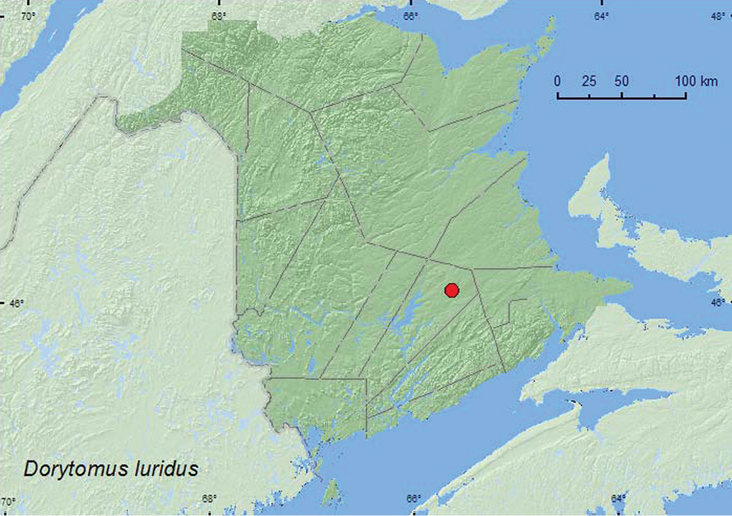
Collection localities in New Brunswick, Canada of *Dorytomus luridus*.

##### Collection and habitat data.

O’Brien (1970) reported *Salix* as the host of this species. The specimen from New Brunswick was captured during July in a Lindgren funnel trap in the canopy of a red oak in an old red oak forest. *Salix* was present nearby along a roadside through the red oak stand.

##### Distribution in Canada and Alaska.

AK, YK, NT, BC, AB, SK, QC, **NB**, NS (McNamara 1991c).

#### Dorytomus marmoreus

Casey, 1892

http://species-id.net/wiki/Dorytomus_marmoreus

Map 21

##### Material examined.

**New Brunswick, Queens Co.**, Cranberry Lake P.N.A., 46.1125°N, 65.6075°W, 20.VII–4.VIII.2011, M. Roy & V. Webster, old red oak forest, Lindgren funnel trap in forest canopy (1, RWC).

**Map 21.**
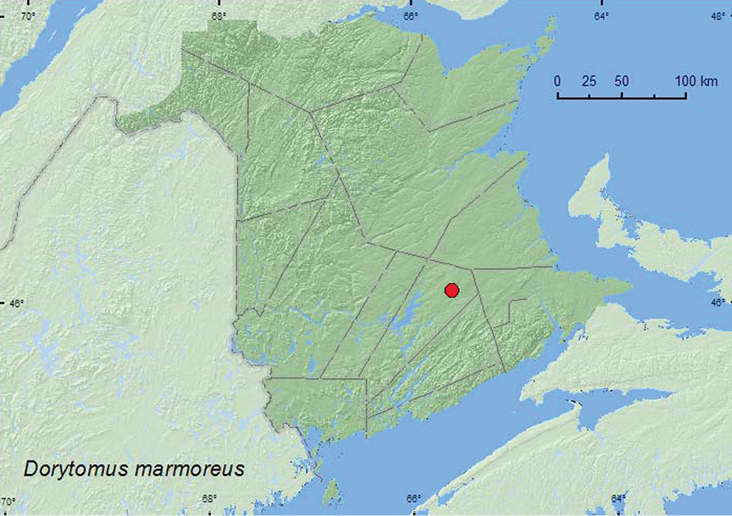
Collection localities in New Brunswick and Quebec. Canada of *Dorytomus marmoreus*.

##### Collection and habitat data.

O’Brien (1970) did not report any host associations for this species. However, hosts of other North American species of *Dorytomus* are either *Salix* or *Populus* (O’Brien 1970). The specimen from New Brunswick was captured between late July and early August in a Lindgren funnel trap in the canopy of a red oak in an old red oak forest. *Salix* and *Populus* were present in or near the red oak stand.

##### Distribution in Canada and Alaska.

AK, AB, ON, QC, **NB**, NS (McNamara 1991c).

### Tribe Mecinini Gistel, 1848

#### Cleopomiarus hispidulus

(LeConte, 1876)**

http://species-id.net/wiki/Cleopomiarus_hispidulus

Map 22

##### Material examined.

**New Brunswick, Queens Co.**, Cranberry Lake P.N.A., 46.1125°N, 65.6075°W, 15–21.VII.2009, R. Webster & M.-A. Giguère, mature red oak forest, Lindgren funnel trap (1, RWC). **York Co.**, Charters Settlement, 45.8430°N, 66.7275°W, 17.VI.2007, R. P. Webster, regenerating mixed forest, sweeping foliage in brushy opening (3, RWC).

**Map 22.**
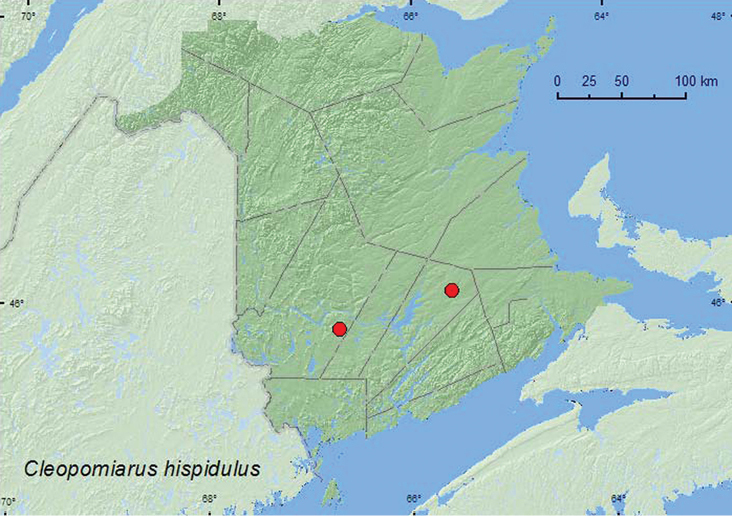
Collection localities in New Brunswick, Canada of *Cleopomiarus hispidulus*.

##### Collection and habitat data.

Larvae of this species feed in seed capsules of *Lobelia* (Campanulaceae) (Anderson 1973). The New Brunswick adults were collected during June and July from a Lindgren trap sample in an old red oak forest and by sweeping foliage in a brushy opening in a regenerating forest.

##### Distribution in Canada and Alaska.

MB, ON, QC, **NB** (McNamara 1991c).

### Tribe Piazorhinini Lacordaire, 1863

#### Piazorhinus pictus

LeConte, 1876

http://species-id.net/wiki/Piazorhinus_pictus

Map 23

##### Material examined.

**New Brunswick, Queens Co.**, Cranberry Lake P.N.A., 46.1125°N, 65.6075°W, 14–19.VIII.2009, R. Webster & M.-A. Giguère, mature red oak forest, Lindgren funnel traps (2, RWC); same locality data and forest type, 20.VII-4.VIII.2011, 4–18.VIII.2011, 18–31.VIII.2011, 31.VIII-15.IX.2011, M. Roy & V. Webster, Lindgren funnel traps (9, AFC, NBM, RWC).

**Map 23.**
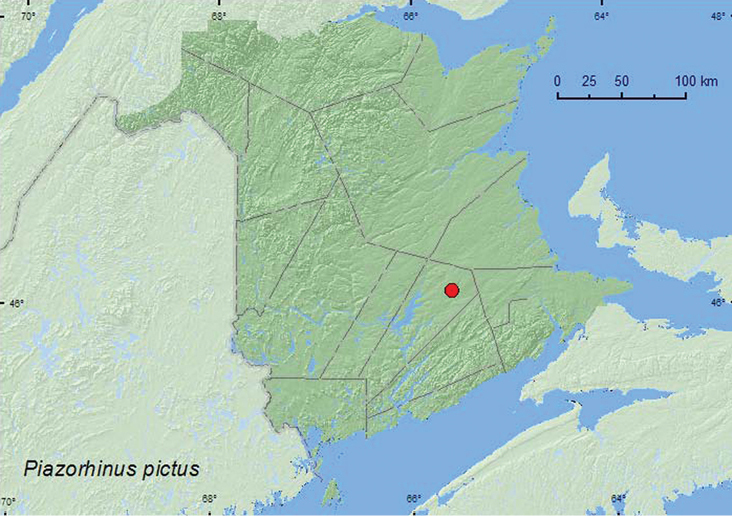
Collection localities in New Brunswick, Canada of *Piazorhinus pictus*.

##### Collection and habitat data.

This species is associated with *Quercus* (Anderson 1993). Adults from New Brunswick were captured during July, August (most during August), and September in Lindgren funnel traps in an old red oak forest.

##### Distribution in Canada and Alaska.

MB, ON, QC, **NB**, NS (McNamara 1991c; Majka et al. 2007b).

### Subfamily Bagoinae Thomson, 1859

***Bagous americanus* LeConte, 1876**

The specimen of *Bagous americanus* reported in Majka et al. (2007b) was misidentified by C. G. Majka and is *Bagous planatus* LeConte. *Bagous americanus* is accordingly removed from faunal list of New Brunswick.

#### Bagous obliquus

LeConte, 1876**

http://species-id.net/wiki/Bagous_obliquus

Map 24

##### Material examined.

**New Brunswick, York Co.**, Charters Settlement, 45.8430°N, 66.7275°W, 17.VI.2007, R. P. Webster, regenerating mixed forest, sweeping foliage in brushy opening (1, RWC).

**Map 24.**
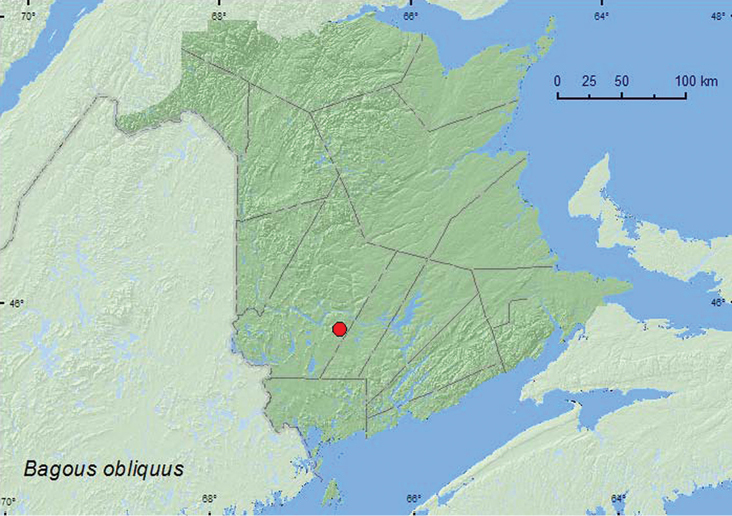
Collection localities in New Brunswick, Canada of *Bagous obliquus*.

##### Collection and habitat data.

*Bagous* spp. are associated with wetland plants such as *Nymphaea* (Nymphaeaceae), *Eleocharis* and *Carex* (Cyperaceae), and *Potamogeton* (Potamogetonaceae) (O’Brien and Marshall 1979).The specimen of *Bagous obliquus*from New Brunswick was swept from foliage in a brushy opening in a regenerating mixed forest (20 years old) near a small marsh in June.

##### Distribution in Canada and Alaska.

ON, QC, **NB** (McNamara 1991c).

#### Bagous planatus

LeConte, 1876**

http://species-id.net/wiki/Bagous_planatus

Map 25

##### Material examined.

**New Brunswick, Charlotte Co.**, near Clark Ridge, 45.3155°N, 67.4406°W, 27.V.2007, R. P. Webster, beaver pond, treading (marsh) vegetation (1, RWC). **Sunbury Co.**, Maugerville, Portobello Creek N.W.A. (National Wildlife Area), 45.8992°N, 66.4248°W, 24.VI.2004, R. P. Webster, silver maple forest, margin of slow river under litter on muddy soil (1, RWC).

**Map 25.**
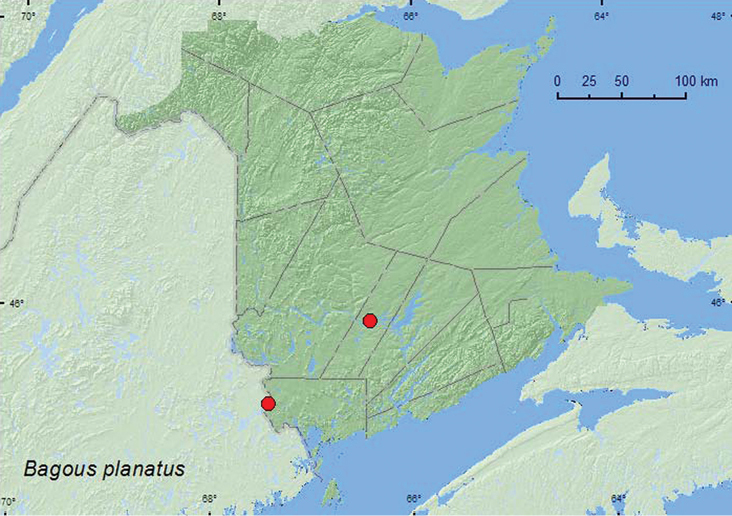
Collection localities in New Brunswick, Canada of *Bagous planatus*.

##### Collection and habitat data.

Adults were collected along the margin of a beaver pond by treading marsh vegetation. Others were found under leaf litter on the margin of a slow flowing river. Adults were captured during May and June.

##### Distribution in Canada and Alaska.

ON, QC, **NB** (McNamara 1991c). Majka et al. (2007b) removed *Bagous planatus* from the faunal list of New Brunswick due to an absence of a supporting voucher specimen. The above records establish this species as a member of the New Brunswick fauna.

### Subfamily Baridinae Schönherr, 1836

**Tribe Apostasimerini Schönherr, 1844**

#### Cylindridia prolixa

(LeConte, 1876)

http://species-id.net/wiki/Cylindridia_prolixa

Map 26

##### Material examined.

**New Brunswick, Gloucester Co.**, Caraquet, near the Acadian Historical Village, 47.7887°N, 65.0756°W, 28.VI.2006, 29.VI.2007, R. P. Webster, salt marsh, on foliage of *Carex paleacea* (13, AFC, RWC).

**Map 26.**
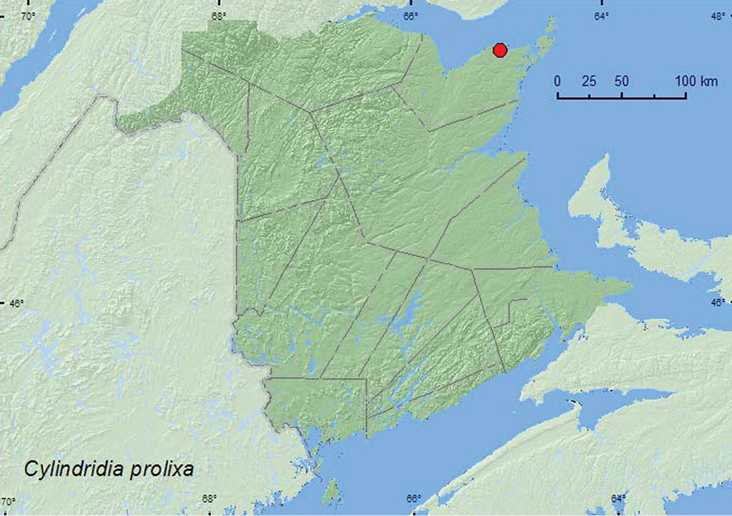
Collection localities in New Brunswick, Canada of *Cylindria prolixa*.

##### Collection and habitat data.

This species is associated with sedges (Cyperaceae) (Anderson 2002). In New Brunswick, adults were collected during June from the foliage of *Carex paleacea* in a salt marsh.

##### Distribution in Canada and Alaska.

MB, ON, QC, **NB**, NS (McNamara 1991c; Majka 2007c).

#### Odontocorynus salebrosus

(Casey, 1892)**

http://species-id.net/wiki/Odontocorynus_salebrosus

Map 27

##### Material examined.

**New Brunswick, York Co.**, Charters Settlement, 45.8340°N, 66.7740°W, 25.VII.2007, R. P. Webster, mature red spruce and red maple forest in old field opening, sweeping foliage (2, RWC).

**Map 27.**
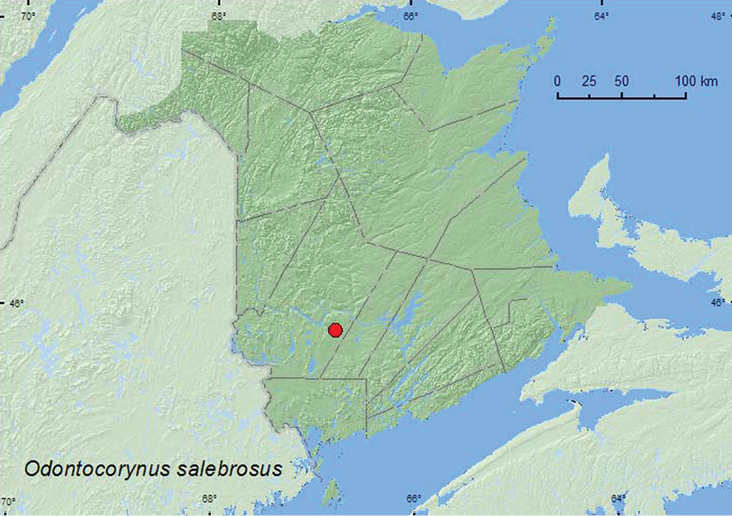
Collection localities in New Brunswick, Canada of *Odontocorynus salebrosus*.

##### Collection and habitat data.

Adults were collected by sweeping an old-field opening in a mixed forest area during July.

##### Distribution in Canada and Alaska.

AB, ON, MB, **NB,** QC, SK (Prena 2008).

### Tribe Baridini Schönherr, 1836

#### Plesiobaris disjuncta

Casey, 1892***

http://species-id.net/wiki/Plesiobaris_disjuncta

Map 28

##### Material examined.

**CANADA, New Brunswick, Carleton Co.**, Belleville, Meduxnekeag Valley Nature Preserve, 46.1888°N, 67.6762°W, 27.VIII.2007, R. P. Webster, upper river margin, sweeping (2, RWC).

**Quebec, Verchères (Co.)**, Varennes, 5.VI.2006, 13.VI.2006, 21.VI.2006, 29.VI.2006, 26.VI.2008, C. Chantal sweeping (5, CCC).

**Map 28.**
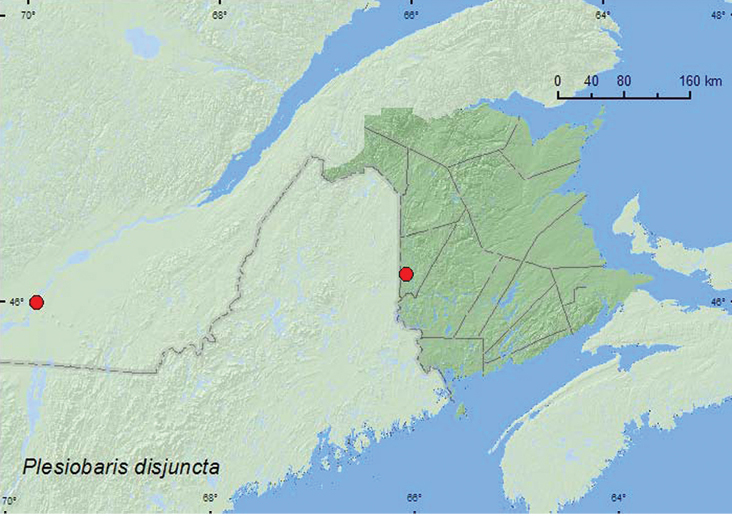
Collection localities in New Brunswick and Quebec, Canada of *Plesiobaris disjuncta*.

##### Collection and habitat data.

Species in this genus are associated with *Hypericum* (Hypericaceae) in wetlands (Anderson 2002). In New Brunswick, adults were collected by sweeping foliage along a river margin during late August. Specimens from Quebec were collected by sweeping during June.

##### Distribution in Canada and Alaska.

**QC, NB** (new Canadian records).

### Subfamily Ceutorhynchinae Gistel, 1848

**Tribe Ceutorhynchini Gistel, 1848**

#### Ceutorhynchus obstrictus

(Marsham, 1802)**

http://species-id.net/wiki/Ceutorhynchus_obstrictus

Map 29

##### Material examined.

**New Brunswick, Carleton Co.**, Bellville, Meduxnekeag Valley Nature Preserve, 46.1890°N, 67.6764°W, 2.VI.2008, R. P. Webster, river margin, on wild mustard (1, RWC). **Gloucester Co.**, Caraquet, near the Acadian Historical Village, 47.7887°N, 65.0756°W, 28.VI.2006, 29.VI.2007, R. P. Webster, inland margin of salt marsh, sweeping (1, RWC).

**Map 29.**
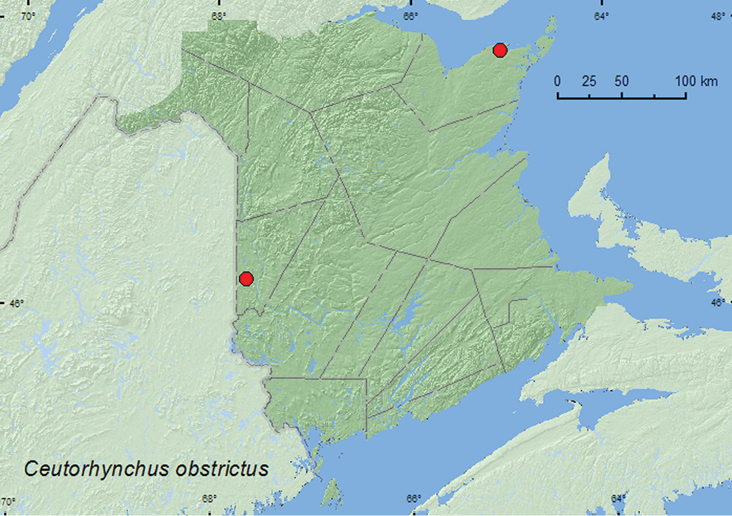
Collection localities in New Brunswick, Canada of *Ceutorhynchus obstrictus*.

##### Collection and habitat data.

Theinvasive*Ceutorhynchus obstrictus*(cabbage seedpod weevil) is a serious pest of canola (*Brassica napus* L.) and oilseed rape (*Brassica rapa* L.) in North America (Cárcamo et al. 2001; Brodeur et al. 2001; Dosdall et al. 2002; Dosdall et al. 2006). In New Brunswick, adults were found on wild mustard on a river margin and swept from foliage on the inland margin of a salt marsh. Adults were collected during June.

##### Distribution in Canada and Alaska.

BC, AB, SK, ON, QC, **NB** (McLeod 1962; Butts and Byers 1996; Brodeur et al. 2001; Dosdall et al. 2002; Mason et al. 2003). The species was first reported (as *Ceutorhynchus assimilis* Paykull) in North America from the the lower mainland of British Columbia, Canada in 1931 (McLeod 1962) and had become well established in Quebec by 2000 (Brodeur et al. 2001).

### Tribe Phytobiini Gistel, 1848

#### Pelenomus sulcicollis

(Fahraeus, 1843)**

http://species-id.net/wiki/Pelenomus_sulcicollis

Map 30

##### Material examined.

**New Brunswick, Saint John Co.**, Chance Harbour, 45.1173°N, 66.3766°W, 28.V.2010, R. P. Webster, salt marsh with sparse grasses & saltwort (glasswort) adjacent to tidal river (1, RWC).

**Map 30.**
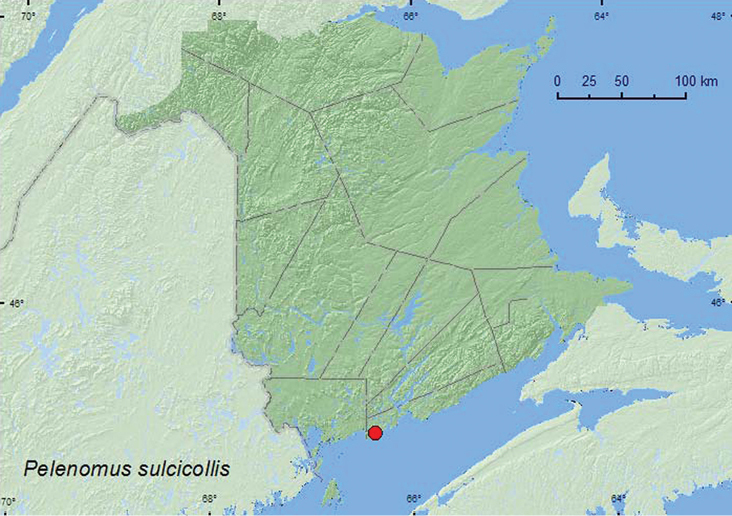
Collection localities in New Brunswick, Canada of *Pelenomus sulcicollis*.

##### Collection and habitat data.

The only specimen from New Brunswick was captured during late May in a salt marsh near a tidal river among sparse grasses and glassworts (*Salicornia europaea* L.).

##### Distribution in Canada and Alaska.

AB, MB, ON, QC, **NB** (McNamara 1991c).

### Subfamily Conoderinae Schönherr, 1833

**Tribe Lechriopini Lacordaire, 1865**

#### Lechriops oculata

(Say, 1824)

http://species-id.net/wiki/Lechriops_oculata

Map 31

##### Material examined.

**New Brunswick, Charlotte Co.**, 10 km NW of New River Beach, 45.2110°N, 66.6170°W, 30.IV–17.V.2010, R. Webster & V. Webster, old growth eastern white cedar forest, Lindgren funnel traps (2, AFC, RWC). **York Co.**, 15 km W of Tracy off Rt. 645, 45.6848°N, 66.8821°W, 16–30.VI.2010, R. Webster & C. MacKay, old red pine forest, Lindgren funnel trap (1, RWC).

**Map 31.**
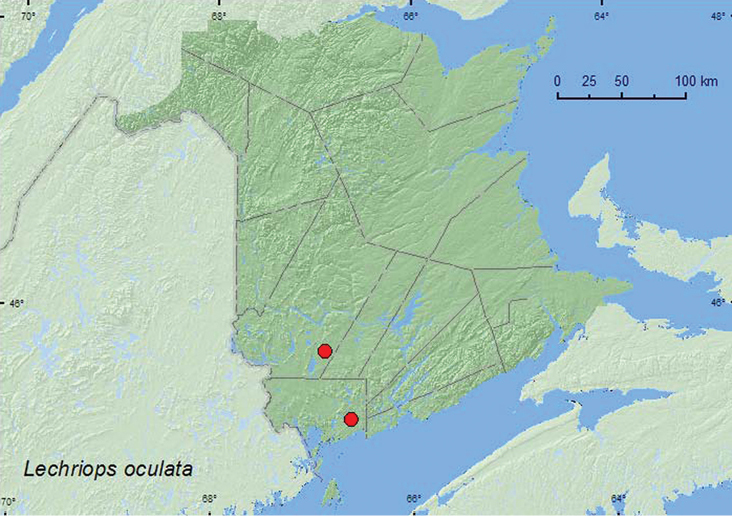
Collection localities in New Brunswick, Canada of *Lechriops oculata*.

##### Collection and habitat data.

This species is associated with hardwood trees, such as oak (*Quercus* sp.), ash (*Fraxinus* sp.), and American beech (*Fagus grandifolia* Ehrh.) (Fagaceae) (Sleeper 1963). In New Brunswick, this species was captured during May and June in Lindgren funnel traps in an old-growth eastern white cedar forest and an old-growth red pine forest. Hardwood species were present at both sites.

##### Distribution in Canada and Alaska.

MB, ON, QC, **NB**, PE, NS (McNamara 1991c; Majka et al. 2007c).

### Tribe Zygopini Lacordaire, 1865

#### Cylindrocopturus longulus

(LeConte, 1876)**

http://species-id.net/wiki/Cylindrocopturus_longulus

Map 32

##### Material examined.

**New Brunswick, York Co.**, 15 km W of Tracy off Rt. 645, 45.6848°N, 66.8821°W, 19–25.V.2009, R. Webster & M.-A. Giguère, old red pine forest, Lindgren funnel trap (1, RWC)

**Map 32.**
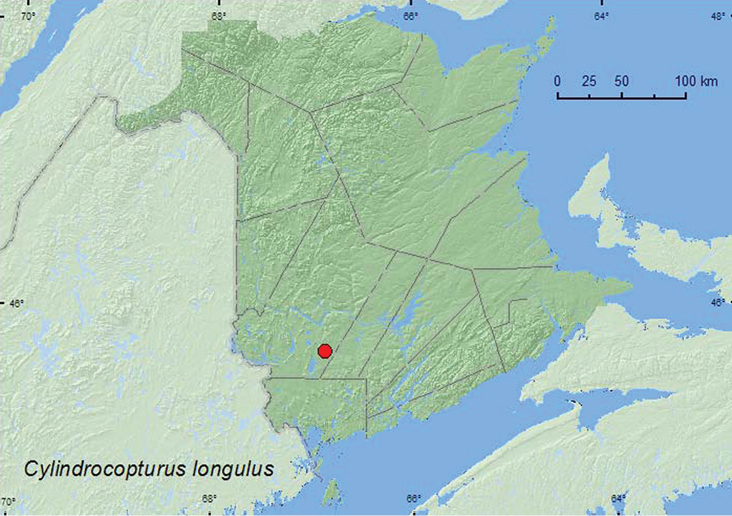
Collection localities in New Brunswick, Canada of *Cylindrocopturus longulus*.

##### Collection and habitat data.

Most species of this genus are associated with Asteraceae, but some are associated with Pinacaeae (Anderson 2002). The single specimen from New Brunswick was captured during May in a Lindgren funnel trap in an old (180-year-old) red pine forest.

##### Distribution in Canada and Alaska.

ON, **NB** (McNamara 1991c).

### Subfamily Cossoninae Schönherr, 1825

**Tribe Cossonini Schönherr, 1825**

#### Cossonus americanus

Buchanan, 1936

http://species-id.net/wiki/Cossonus_americanus

Map 33

##### Material examined.

**New Brunswick, Queens Co.**, Cranberry Lake P.N.A., 46.1125°N, 65.6075°W, 7–22.VI.2011, 29.VI–7.VII.2011, 13–20.VII.2011, M. Roy & V. Webster, old red oak forest, Lindgren funnel traps (3, NBM, RWC). **York Co.**, 15 km W of Tracy off Rt. 645, 45.6848°N, 66.8821°W, 21–28.VI.2009, R. Webster & M.-A. Giguère, old red pine forest, Lindgren funnel trap (1, RWC); same locality and forest type but 16–30.VI.2010, R. Webster & C. MacKay, Lindgren funnel traps (2, AFC, RWC).

**Map 33.**
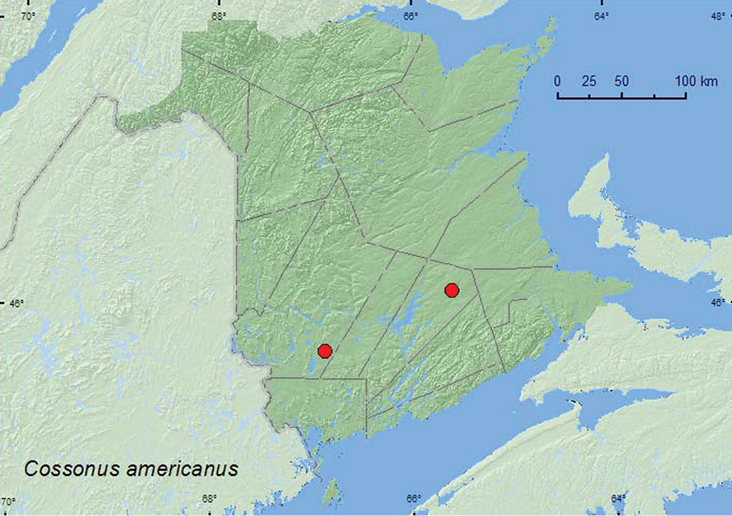
Collection localities in New Brunswick, Canada of *Cossonus americanus*.

##### Collection and habitat data.

O’Brien (1997) reported *Populus balsamifera* as a host for this species. Specimens of *Cossonus americanus* were captured during June and July in Lindgren funnel traps in an old red pine forest and an old red oak forest.

##### Distribution in Canada and Alaska.

QC, **NB**, NS, NF (McNamara 1991c).

### Tribe Onycholipini Wollaston, 1873

#### Stenoscelis brevis

(Boheman, 1845)

http://species-id.net/wiki/Stenoscelis_brevis

Map 34

##### Material examined.

**New Brunswick, Queens Co.**, Grand Lake Meadows P.N.A., 45.8227°N, 66.1209°W, 29.VI–12.VII.2010, 12–26.VII.2010, 26.VII–7.VIII.2010, R. Webster, C. MacKay, M. Laity & R. Johns, old silver maple forest with green ash and seasonally flooded marsh, Lindgren funnel traps (45, AFC, RWC); same locality data and forest type, 5–19.VII.2011, 19.VII–5.VIII.2011, M. Roy & V. Webster, Lindgren funnel traps (7, NBM, RWC).

**Map 34.**
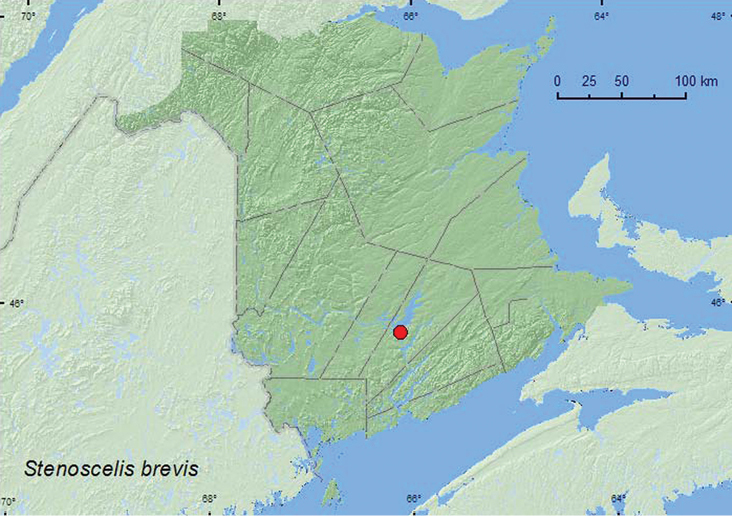
Collection localities in New Brunswick, Canada of *Stenoscelis brevis*.

##### Collection and habitat data.

This species occurs under bark or in dead wood of a variety of hardwood species such as apple, hawthorn (*Crataegus*), elm (*Ulmus*), ash, maple (*Acer*), and oak (O’Brien 1997). In New Brunswick, a large series of adults was captured during July and August in Lindgren funnel traps in a silver maple swamp.

##### Distribution in Canada and Alaska.

ON, QC, **NB**, NS (McNamara 1991c; Majka et al. 2007c).

### Tribe Rhyncolini Gistel, 1848

#### Himatium errans

LeConte, 1876

http://species-id.net/wiki/Himatium_errans

Map 35

##### Material examined.

**New Brunswick, Sunbury Co.**, Acadia Research Forest, 45.9866°N, 66.3841°W, 8–13.VII.2009, R. Webster & M.-A. Giguère, red spruce forest with red maple and balsam fir, Lindgren funnel trap (1, RWC). **York Co.**, 15 km W of Tracy off Rt. 645, 45.6848°N, 66.8821°W, 29.VII-4.VIII.2009, R. Webster & M.-A. Giguère, old red pine forest, Lindgren funnel trap (1, RWC); same locality and forest type but 4–16.VI.2010, 16–30.VI.2010, 30.VI–13.VII.2010, 13–27.VII.2010, R. Webster & C. MacKay, Lindgren funnel traps (6, AFC, RWC); 14 km WSW of Tracy, S of Rt. 645, 45.6741°N, 66.8661°W, 2–16.VI..2010, R. Webster & C. MacKay, old mixed forest with red and white spruce, red and white pine, balsam fir, eastern white cedar, red maple, and *Populus* sp., Lindgren funnel trap (1, RWC).

**Map 35.**
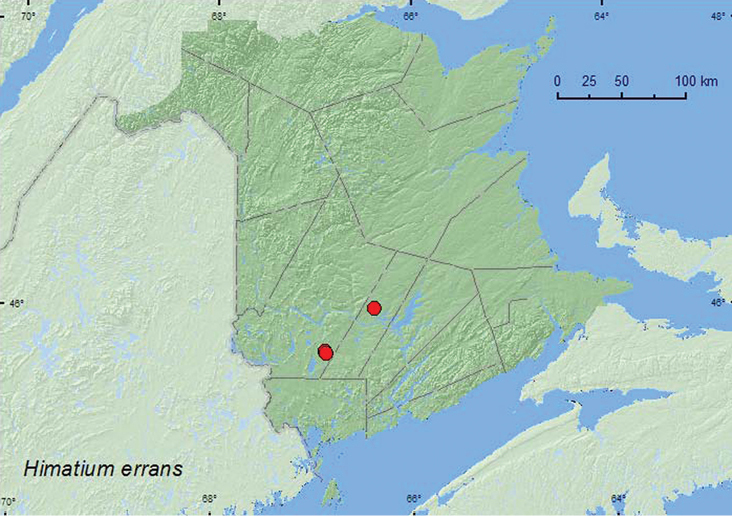
Collection localities in New Brunswick, Canada of *Himatium errans*.

##### Collection and habitat data.

This species was reported from *Ips* galleries in *Pinus*, and emerged indoors from an *Acer saccharinum* branch (O’Brien 1997). In New Brunswick, *Himatium errans* was captured in June, July, and August in Lindgren funnel traps in an old red pine forest, a red spruce (*Picea rubens* Sarg.) forest, and an old mixed forest.

##### Distribution in Canada and Alaska.

QC, **NB**, NS (McNamara 1991c).

#### Phloeophagus apionides

Horn, 1873

http://species-id.net/wiki/Phloeophagus_apionides

Map 36

##### Material examined.

**New Brunswick, Carleton Co.**, Jackson Falls, Bell Forest, 46.2200°N, 67.7231°W, 5–12.VII.2008, R. P. Webster, mature hardwood forest, Lindgren funnel traps (2, AFC, RWC). **Charlotte Co.**, 10 km NW of New River Beach, 45.2110°N, 66.6170°W, 15–29.VI.2010, R. Webster & C. MacKay, old growth eastern white cedar forest, Lindgren funnel trap (1, RWC). **Queens Co.**, Cranberry Lake P.N.A., 46.1125°N, 65.6075°W, 10–15.VII.2009, 15–21.VII.2009, R. Webster & M.-A. Giguère, old red oak forest, Lindgren funnel traps (2, AFC, RWC); Grand Lake Meadows P.N.A., 45.8227°N, 66.1209°W, 31.V–15.VI.2010, R. Webster & C. MacKay, old silver maple forest with green ash and seasonally flooded marsh, Lindgren funnel traps (3, NBM, RWC); same locality data and forest type, 19.VII-–5.VIII.2011, M. Roy & V. Webster, Lindgren funnel trap (1, NBM). **Restigouche, Co.**, Dionne Brook P.N.A., 47.9064°N, 68.3441°W, 27.VI–14.VII.2011, M. Roy & V. Webster, old-growth white spruce and balsam fir forest, Lindgren funnel trap (1, NBM). **York Co.**, 15 km W of Tracy off Rt. 645, 45.6848°N, 66.8821°W, 16–30.VI.2010, 13–27.VII.2010, R. Webster & C. MacKay, old red pine forest, Lindgren funnel traps (2, RWC); 14 km WSW of Tracy, S of Rt. 645, 45.6741°N, 66.8661°W, 2–16.VI..2010, 16–30.VI.2010, R. Webster & C. MacKay, old mixed forest with red and white spruce, red and white pine, balsam fir, eastern white cedar, red maple, and *Populus* sp., Lindgren funnel traps (2, AFC, RWC).

**Map 36.**
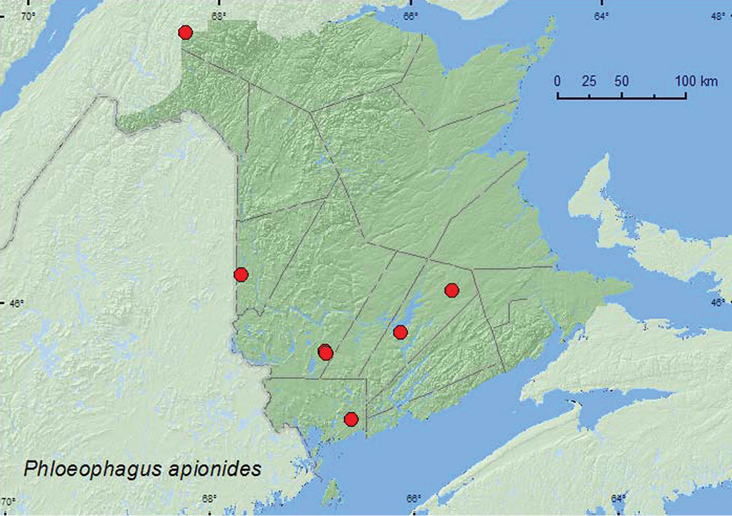
Collection localities in New Brunswick, Canada of *Phloeophagus apionides*.

##### Collection and habitat data.

Adults were captured during June, July, and August in Lindgren funnel traps in a hardwood forest, an eastern white cedar forest, an old red oak forest, an old silver maple forest, an old-growth red pine forest, an old-growth northern hardwood forest, and an old mixed forest. This species is associated with hardwood trees such as wild cherry (*Prunus* sp.), ash, and white oak (O’Brien 1997).

##### Distribution in Canada and Alaska.

ON, QC, **NB**, NS (McNamara 1991c; Majka et al. 2007c).

#### Phloeophagus canadensis

Van Dyke, 1927**

http://species-id.net/wiki/Phloeophagus_canadensis

Map 37

##### Material examined.

**New Brunswick, Carleton Co.**, Jackson Falls, Bell Forest, 46.2200°N, 67.7231°W, 19–27.VI.2008, R. P. Webster, mature hardwood forest, Lindgren funnel trap (1, RWC). **Queens Co.**, Grand Lake Meadows P.N.A., 45.8227°N, 66.1209°W, 2–21.VI.2011, 27.VI–5.VII.2011, M. Roy & V. Webster, old silver maple forest and seasonally flooded marsh, Lindgren funnel traps (3, RWC); Cranberry Lake P.N.A., 46.1125°N, 65.6075°W, 7–22.VI.2011, M. Roy & V. Webster, old red oak forest, Lindgren funnel trap (1, RWC). **Restigouche Co.**, Dionne Brook P.N.A., 47.9030°N, 68.3503°W, 27.VI–14.VII.2011, 14–28.VII.2011, M. Roy & V. Webster, old-growth northern hardwood forest, Lindgren funnel traps (2, RWC). **York Co.**, 14 km WSW of Tracy, S of Rt. 645, 45.6741°N, 66.8661°W, 2–16.VI.2010, R. Webster & C. MacKay, old mixed forest with red and white spruce, red and white pine, balsam fir, eastern white cedar, red maple, and *Populus* sp., Lindgren funnel trap (1, RWC).

**Map 37.**
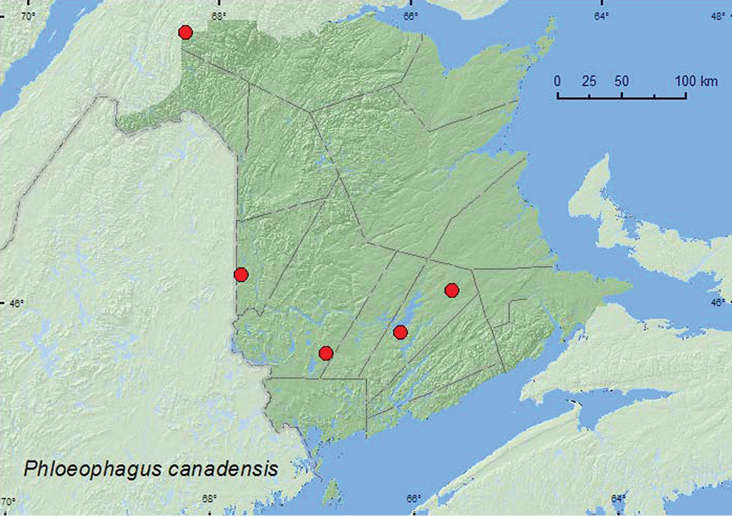
Collection localities in New Brunswick, Canada of *Phloeophagus canadensis*.

##### Collection and habitat data.

O’Brien (1997) reported *Populus* as a host of *Phloeophagus canadensis*. The New Brunswick adults were captured during June and July in Lindgren funnel traps in a hardwood forest, an old mixed forest, an old red oak forest, an old-growth northern hardwood forest, and an old silver maple forest. *Populus* was present at all the sites where this species was captured.

##### Distribution in Canada and Alaska.

BC, AB, MB, QC, **NB** (McNamara 1991c).

#### Phloeophagus minor

Horn, 1873**

http://species-id.net/wiki/Phloeophagus_minor

Map 38

##### Material examined.

**New Brunswick, Queens Co.**, Grand Lake Meadows P.N.A., 45.8227°N, 66.1209°W, 29.VI-12.VII.2010, R. Webster, C. MacKay, M. Laity, & R. Johns, old silver maple forest with green ash and seasonally flooded marsh, Lindgren funnel traps (2, RWC); same locality data and forest type, 5–19.VII.2011, M. Roy & V. Webster, Lindgren funnel traps (2, RWC).

**Map 38.**
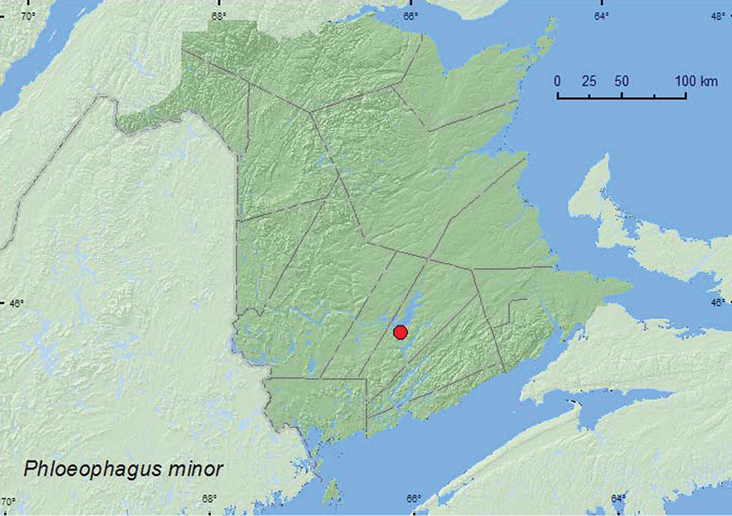
Collection localities in New Brunswick, Canada of *Phloeophagus minor*.

##### Collection and habitat data.

O’Brien (1997) reported this species as occurring in decaying trunks of various hardwood species such as birch (*Betula* sp.), willow (*Salix* sp.), and elm, and on dead twigs. The specimens from New Brunswick were captured during July in Lindgren funnel traps in a silver maple swamp.

##### Distribution in Canada and Alaska.

ON, QC, **NB** (McNamara 1991c).

### Subfamily Cyclominae Schönherr, 1826

**Tribe Listroderini LeConte, 1876**

#### Listronotus deceptus

(Blatchley, 1916)**

http://species-id.net/wiki/Listronotus_deceptus

Map 39

##### Material examined.

**New Brunswick, Gloucester Co.**, Caraquet, near the Acadian Historical Village, 47.7887°N, 65.0756°W, 29.VI.2007, R. P. Webster, inland margin of salt marsh, sweeping (4, RWC).

**Map 39.**
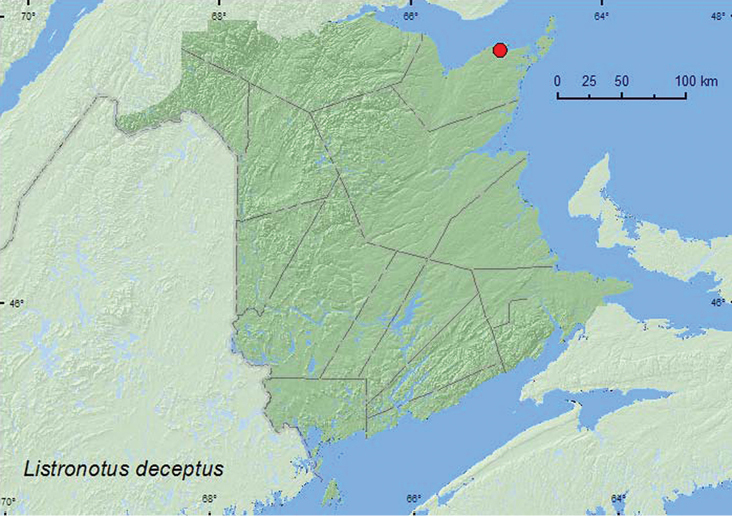
Collection localities in New Brunswick, Canada of *Listronotus deceptus*.

##### Collection and habitat data.

Little is known about the habitat associations or biology of this species. Adults from New Brunswick were swept from foliage on the inland margin of a salt marsh during late June.

##### Distribution in Canada and Alaska.

QC, **NB** (O’Brien 1997).

#### Listronotus lutulentus

(Boheman, 1843)**

http://species-id.net/wiki/Listronotus_lutulentus

Map 40

##### Material examined.

**New Brunswick, Sunbury Co.**, near Sunpoke Lake, 45.7662°N, 66.5526°W, 20.VI.2007, 28.VII.2007, 10.VII.2008, R. P. Webster, seasonally flooded marsh, sweeping *Sagittaria* species (6, RWC).

**Map 40.**
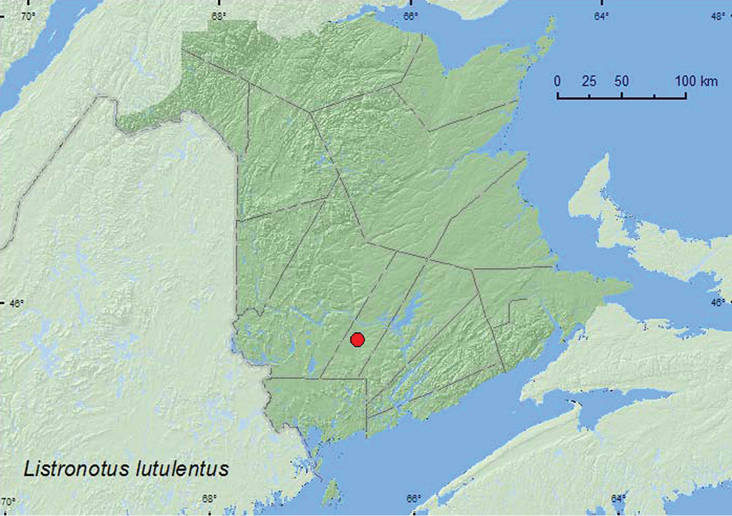
Collection localities in New Brunswick, Canada of *Listronotus lutulentus*.

##### Collection and habitat data.

*Listronotus lutulentus* has been swept from sedges (O’Brien 1997), otherwise little is known about the biology of this species. Adults from New Brunswick were swept from *Sagittaria* sp. in a seasonally flooded marsh during June and July.

##### Distribution in Canada and Alaska.

MB, ON, QC, **NB** (McNamara 1991c).

#### Listronotus oregonensis

(LeConte, 1876)

http://species-id.net/wiki/Listronotus_oregonensis

Map 41

##### Material examined.

**New Brunswick, Gloucester Co.**, East Allardville (Allardville East), 10.VI.1942, W. Raiche, on *Abies balsamea*, beating, F.I.S., 42–1-37 (1, AFC). **Queens Co.**, Grand Lake Meadows P.N.A., 45.8227°N, 66.1209°W, 5–19.VII.2011, M. Roy & V. Webster, old silver maple forest and seasonally flooded marsh, Lindgren funnel trap (1, RWC). **York Co.** Mazerolle Settlement, 45.8729°N, 66.8311°W, 28.IV.2006, R. P. Webster, stream margin (in beaver meadow), on mud with sparse vegetation (1, RWC).

**Map 41.**
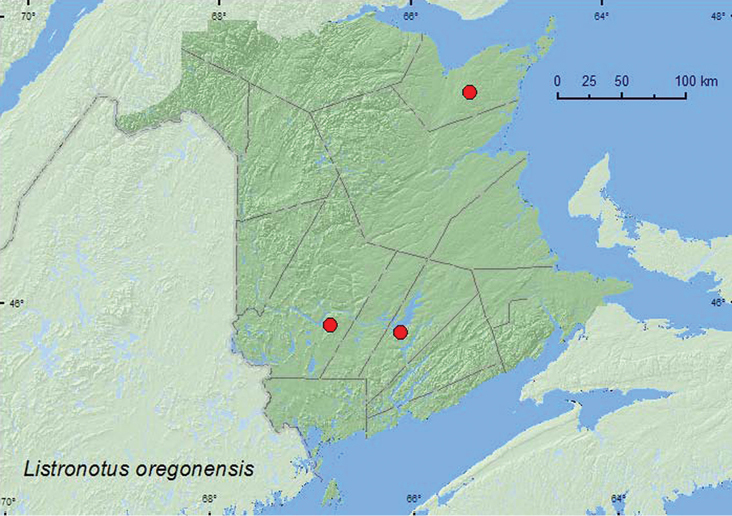
Collection localities in New Brunswick, Canada of *Listronotus oregonensis*.

##### Collection and habitat data.

This species, known as the carrot weevil or parsley weevil, is associated with various species of Apiaceae, *Plantago* spp., and *Rumex* spp. (O’Brien 1997; Torres and Hoy 2002). In New Brunswick, one adult was beaten from balsam fir (*Abies balsamea* (L.) Mill.) (probably incidental), another on mud along a stream margin in a beaver meadow, and one adult was captured in a Lindgren funnel trap in an old silver maple swamp. Adults were collected during April, June, and July.

##### Distribution in Canada and Alaska.

MB, ON, QC, **NB**, PE, NS (McNamara 1991c; Majka et al. 2007c).

### Subfamily Hyperinae Marseul, 1863

**Tribe Hyperini Marseul, 1863**

#### Hypera compta

(Say, 1831)**

http://species-id.net/wiki/Hypera_compta

Map 42

##### Material examined.

**New Brunswick, Queens Co.**, Grand Lake near Scotchtown, 45.8762°N, 66.1816°W, 3.VI.2007, R. P. Webster, red oak and maple forest near lakeshore, sweeping foliage (1, RWC); W of Jemseg at “Trout Creek”, 45.8237°N, 66.1225°W, 6.IX.2007, R. P. Webster, silver maple swamp, sweeping foliage along margin of marsh (2, RWC).

**Map 42.**
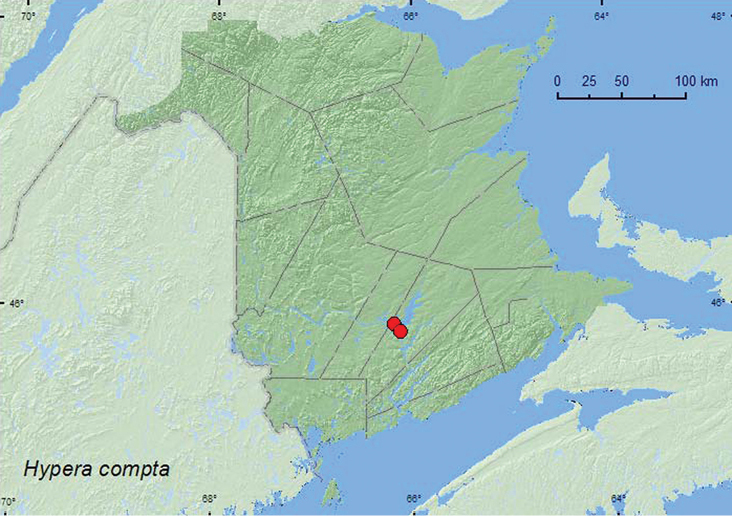
Collection localities in New Brunswick, Canada of *Hypera compta*.

##### Collection and habitat data.

Adults were swept from foliage near a lakeshore and the margin of a seasonally flooded marsh near a silver maple swamp during June and September.

##### Distribution in Canada and Alaska.

BC, ON, QC, **NB** (McNamara 1991c).

### Subfamily Lixinae Schönherr, 1823

**Tribe Lixini Schönherr, 1825**

#### Lixus rubellus

Randall, 1838**

http://species-id.net/wiki/Lixus_rubellus

Map 43

##### Material examined.

**New Brunswick, Carleton Co.**, Bellville, Meduxnekeag Valley Nature Preserve, 46.1931°N, 67.6825°W, 8.VI.2008, R. P. Webster, flood plain forest, on flowers of *Crataegus* species (1, RWC). **Queens Co.**, Grand Lake near Scotchtown, 45.8762°N, 66.1816°W, 3.VI.2007, R. P. Webster, red oak and maple forest near lakeshore, beating foliage of *Amelanchier* species (4, RWC).

**Map 43.**
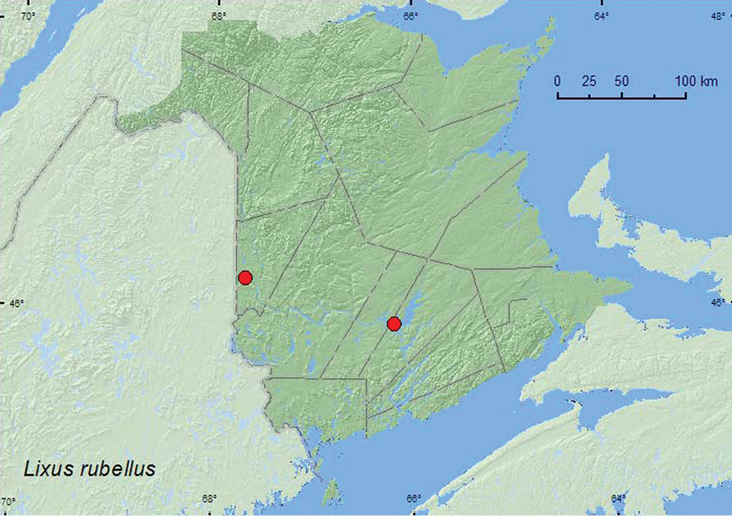
Collection localities in New Brunswick, Canada of *Lixus rubellus*.

##### Collection and habitat data.

Adults of the genus *Lixus* are associated with members of the families Asteraceae and Polygonaceae (Anderson 2002). In New Brunswick, adults were collected in June from flowers of *Crataegus* along a river margin and beating foliage of *Amelanchier* near a lakeshore.

##### Distribution in Canada and Alaska.

NT, BC, AB, SK, MB, ON, QC, **NB** (McNamara 1991c).

### Subfamily Mesoptiliinae Lacordaire, 1863

**Tribe Magdalidini Pascoe, 1870**

#### Magdalis alutacea

LeConte, 1878**

http://species-id.net/wiki/Magdalis_alutacea

Map 44

##### Material examined.

**New Brunswick, Albert Co.**, Hillsborough, 13.VII.1966, R.G. Carlin, on fir, F.I.S. 66–2078–01 (1, AFC). **Charlotte Co.**, Deer Island Point, Lambertville, 10.VII.1939, H. M. Lambert, conifer forest, on *Picea* sp., F.I.S., 39-L211 (1, AFC). **Gloucester Co.**, Allardville, Lord Foy Brook, 24.VI.1940, Albany Morais, on fir, beating, F.I.S., 40-L97 (1, AFC). **Kings Co.**, Grays Mills, 17.V.1921, 1.VI.1921, R.P.G. (7, AFC); 2 mi W of Hampton near Pickwaket Rd., 25.VI.1961, (no collector given), ex. white spruce, beating, F.I.S., 61–0621 (1, AFC). **Queens Co.**, Cherryvale, 15.VI.1964, D.R. Edling, conifer forest, ex. red spruce, beating, F.I.S., 64–0529–07 (1, AFC); **Sunbury Co.**, Acadia Forest Experiment Station, 12.VI.1987, (no collector given) black spruce, ARNEWS plot 201, 87–2-2149–03 (1, AFC); Acadia Research Forest, 45.9866°N, 66.3841°W, 4–11.VIII.2009, R. Webster & M.-A. Giguère, mature (110-year-old) red spruce forest with scattered red maple and balsam fir, Lindgren funnel trap (1, AFC). **York Co.**, 15 km W of Tracy off Rt. 645, 45.6837°N, 66.8805°W, 26.VI.2007, R. P. Webster, old red pine forest, on foliage of *Pinus strobus* (1, RWC).

**Map 44.**
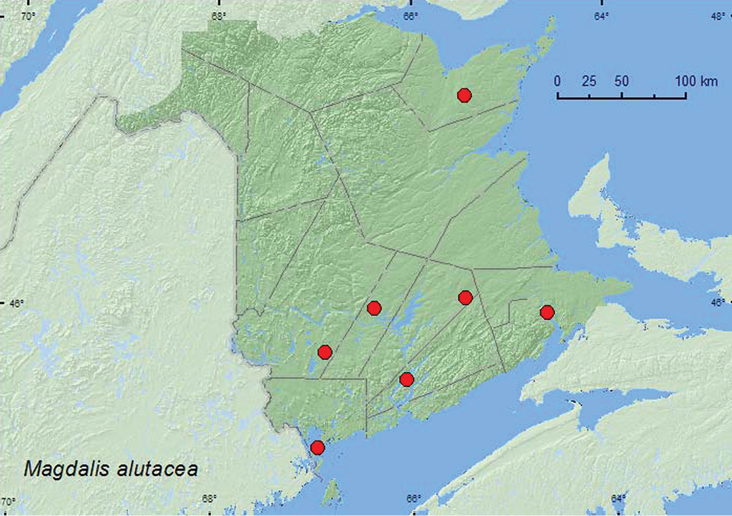
Collection localities in New Brunswick, Canada of *Magdalis alutacea*.

##### Collection and habitat data.

In New Brunswick, adults were collected (beating) from foliage of *Picea* spp. (red spruce, white spruce (*Picea glauca* (Moench) Voss), and black spruce (*Picea mariana* (Mill.) B.S.P.)), balsam fir, and white pine (*Pinus strobus* L.). One individual was captured in a Lindgren funnel trap in a mature red spruce forest. Adults were collected during June, July and August.

##### Distribution in Canada and Alaska.

AK, YK, NT, BC, AB, QC, **NB** (McNamara 1991c).

#### Magdalis barbita

(Say, 1831)

http://species-id.net/wiki/Magdalis_barbita

Map 45

##### Material examined.

**New Brunswick, Kings Co.**, 2 mi N of Norton, 4.IX.1968 (larval collection date), emerged 12.VI.1969, MacCall, reared from white elm, F.I.S., 68–2-3492–01 (2, AFC). **Queens Co.**, Welsford, 25.V.1962 (pupal collection date), emerged 28.V.1962, 29.V.1962, 29.V.1962, 4.VI.1962, 6.VI.1962, 14.VI.1962, 19.VI.1962, C. C. Smith, under bark of white elm, F.I.S., 62–0083 (17, AFC); Waterborough, Wiggins Cove, 19.VI.1968, MacCall, under bark of white elm, F.I.S., 68–3528–02 (2, AFC); Grand Lake Meadows P.N.A., 45.8227°N, 66.1209°W, 3–21.VI.2011, 21.VI-5.VII.2011, M. Roy & V. Webster, old silver maple forest with green ash and seasonally flooded marsh, Lindgren funnel traps deployed in forest canopy (2, RWC). **Westmorland Co.**, Moncton, McLaughlin Rd., 18.IX.1968 (larval collection date), emerged 18.VI.1969, MacCall, ex. *Ulmus americana*, F.I.S., 68–2-3727–01 (1, AFC). **York Co.**, Fredericton, emerged 22.III.1950, 31.III.1950, (no collector given), reared from elm (4, AFC); Fredericton, York St., 29.VII.1968 (larval collection date), emerged. 3.VII.1970, (no collector given), reared from white elm, F.I.S., 69–2-2278–01 (3, AFC); 2 km S of Tay Mills off Rt. 620 at South Tay Bridge, 28.VIII.1959 (host collection date), emerged 1. VI.1960, Moran, emerged from white elm, F.I.S., 59–1561 (11, AFC); Millville, (no collector given) reared from elm (5, AFC); Forest City, emerged 4.III.1969, 12.III.1969, 28.III.1969, (no collector given) reared from white elm, F.I.S., 68–2-4024–03 (4, AFC).

**Prince Edward Island, Prince Co.,** Woodstock, 17.VI.1969, MacCall, on young foliage of white elm, F.I.S., 69–2-1063–04 (1, AFC).

**Map 45.**
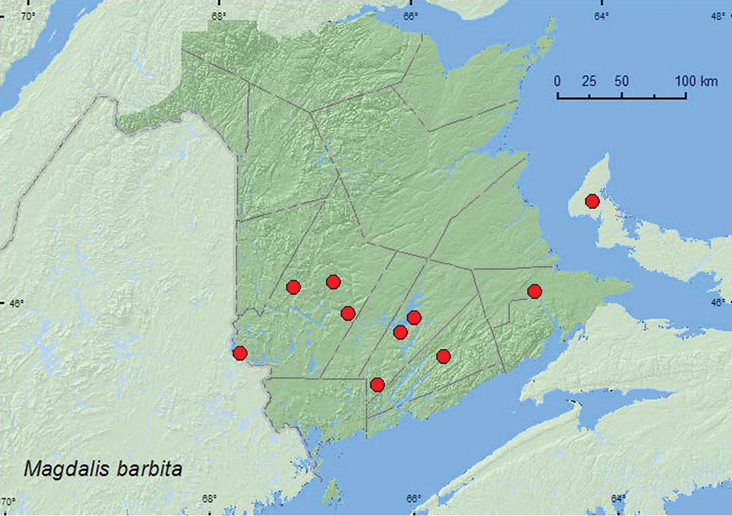
Collection localities in New Brunswick and Prince Edward Island, Canada of *Magdalis barbita*.

##### Collection and habitat data.

*Magdalis barbita* breeds in the trunks and branches of unhealthy *Ulmus* spp. (Drooz 1985) and is also reported to be associated with *Quercus* and *Carya* (Juglandaceae) (Blatchley and Leng 1916). Most adults from New Brunswick were reared from larvae or pupae collected from under barkof American or white elm (*Ulmus americana* L.). Adults were captured during June and July in Lindgren funnel trap in an old silver maple swamp. *Ulmus americana* was present near the trap. The adult from Prince Edward Island was collected from young foliage of *Ulmus americana* during June.

##### Distribution in Canada and Alaska.

MB, ON, QC, **NB**, NS, **PE** (McNamara 1991c; Majka et al. 2007c).

#### Magdalis hispoides

LeConte, 1876**

http://species-id.net/wiki/Magdalis_hispoides

Map 46

##### Material examined.

**New Brunswick, Carleton Co.**, Lindsay, 4.VII.1963, B. Denny, ex. balsam fir, beating, F.I.S. 63–0860–04 (1, AFC). **Queens Co.**, Cherryvale, 15.VI.1964, D. R. Edling, conifer forest, ex. red spruce, beating, F.I.S., 64–0529–07 (1, AFC). **Victoria Co.**, Hazeldean, 17.VI.1963, (no collector given), ex. trembling aspen, beating, F.I.S., 63–0544–02 (1, AFC).

**Map 46.**
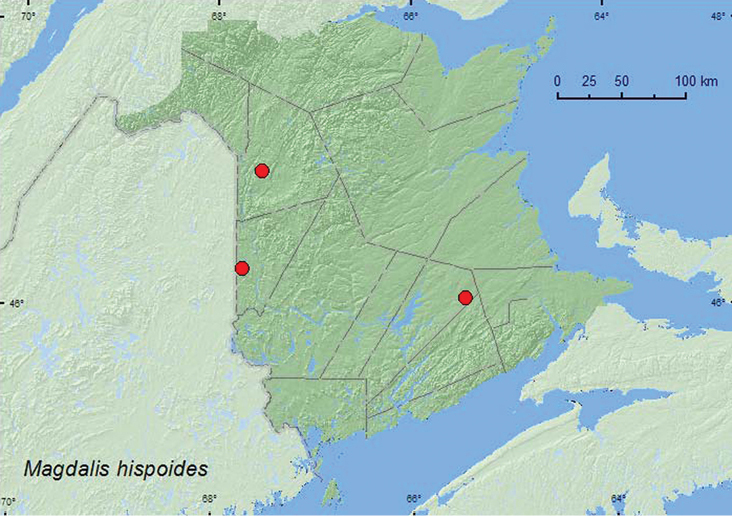
Collection localities in New Brunswick, Canada of *Magdalis hispoides*.

##### Collection and habitat data.

*Magdalis hispoides* adults have been observed feeding on needles of *Pinus strobus* (Plumb 1950). In New Brunswick, adults were beaten from foliage of balsam fir, red spruce, and trembling aspen during June and July.

##### Distribution in Canada and Alaska.

YK, BC, AB, ON, QC, **NB**, NF (McNamara 1991c).

#### Magdalis perforata

Horn, 1873

http://species-id.net/wiki/Magdalis_perforata

Map 47

##### Material examined.

**New Brunswick, York Co.**, 15 km W of Tracy off Rt. 645, 45.6848°N, 66.8821°W, 4–16.VI.2010, 30.VI–13.VII.2010, 27.VII–10.VIII.2010, R. Webster, K. Burgess, C. Hughes & C. MacKay, old red pine forest, Lindgren funnel trap (3, AFC, RWC).

**Map 47.**
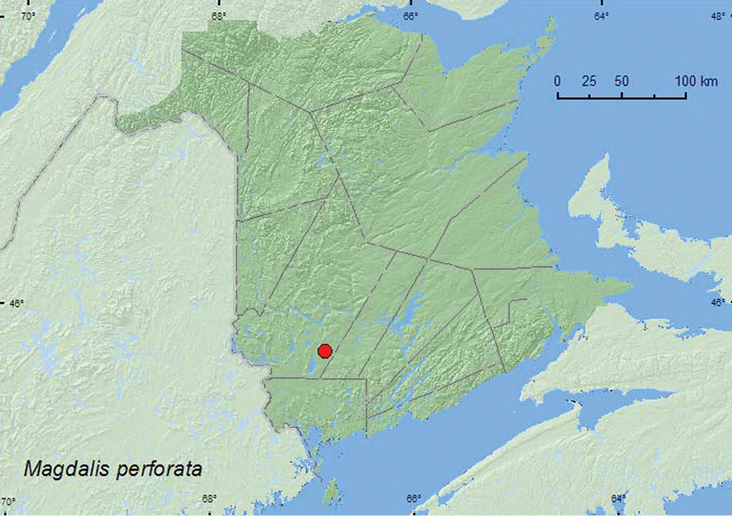
Collection localities in New Brunswick, Canada of *Magdalis perforata*.

##### Collection and habitat data.

*Magdalis perforata* breeds in dead and dying branches of pines (Martin 1964). In New Brunswick, adults were captured during June, July, and, August in Lindgren funnel traps in an old red pine forest.

##### Distribution in Canada and Alaska.

ON, QC, **NB**, NS (McNamara 1991c; Majka et al. 2007c).

### Subfamily Molytinae Schönherr, 1823

**Tribe Conotrachelini Jekel, 1865**

#### Conotrachelus juglandis

LeConte, 1876**

http://species-id.net/wiki/Conotrachelus_juglandis

Map 48

##### Material examined.

**New Brunswick, Carleton Co.**, Jackson Falls, Bell Forest, 46.2200°N, 67.7231°W, 13.VIII.2007, R. P. Webster, mature hardwood forest (with butternut), sweeping foliage (1, RWC); same locality and habitat but 28.IV–9.V.2009, 1–8.VI.2009, R. Webster & M.-A. Giguère, Lindgren funnel traps (3, AFC, RWC); Bellville, Meduxnekeag Valley Nature Preserve, 46.1931°N, 67.6825°W, 8.VI.2008, R. P. Webster, floodplain forest (with butternut), on flowers of *Prunus virginiana* (beating) (1, RWC); same locality and collector but 46.1930°N, 67.6821°W, 13.VII.2008, floodplain forest (with butternut), sweeping foliage (1, RWC).

**Map 48.**
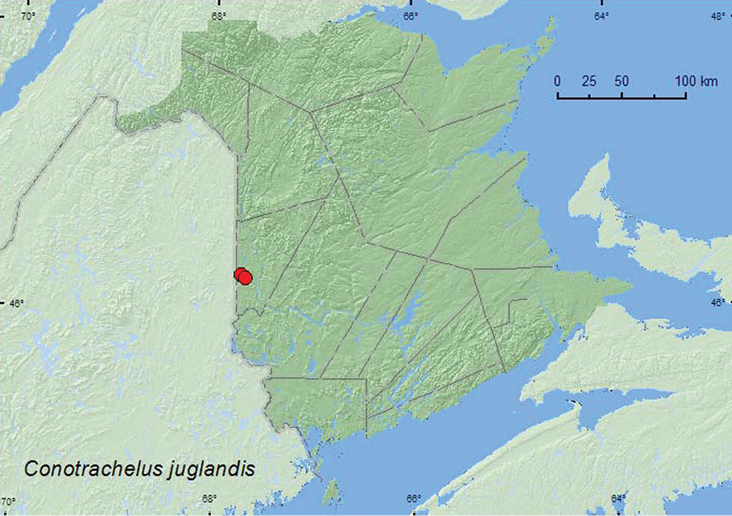
Collection localities in New Brunswick, Canada of *Conotrachelus juglandis*.

##### Collection and habitat data.

*Conotrachelus juglandis* breeds in the nuts, stems, and leaf petioles of *Juglans* spp. (Drooz 1985). Most adults from New Brunswick were swept from foliage in hardwood forests and floodplain forests with butternut (*Juglans cinerea* L.). One individual was beaten from flowers of choke cherry (*Prunus virginiana* L.). A few adults were captured in Lindgren funnel traps in a hardwood forest with butternut. Adults were collected during April, June, and August.

##### Distribution in Canada and Alaska.

ON, QC, **NB** (McNamara 1991c).

#### Conotrachelus posticatus

Boheman, 1837

http://species-id.net/wiki/Conotrachelus_posticatus

Map 49

##### Material examined.

**New Brunswick, Queens Co.**, Cranberry Lake P.N.A., 46.1125°N, 65.6075°W,, 12–21.V.2009, 21–27.V.2009, 27.V-5.VI.2009, 11–18.VI.2009, 18–25.VI.2009, 25.VI–1.VII.2009, R. Webster & M.-A. Giguère, mature red oak forest, Lindgren funnel traps, (a few individuals were swept from foliage) (20, AFC, RWC). **Restigouche Co**., Jacquet River Gorge P.N.A., 47.8111°N, 65.9945°W,, 17.VIII.2010, A. Fairweather & K. Vandenbroeck (1, NBM).

**Map 49.**
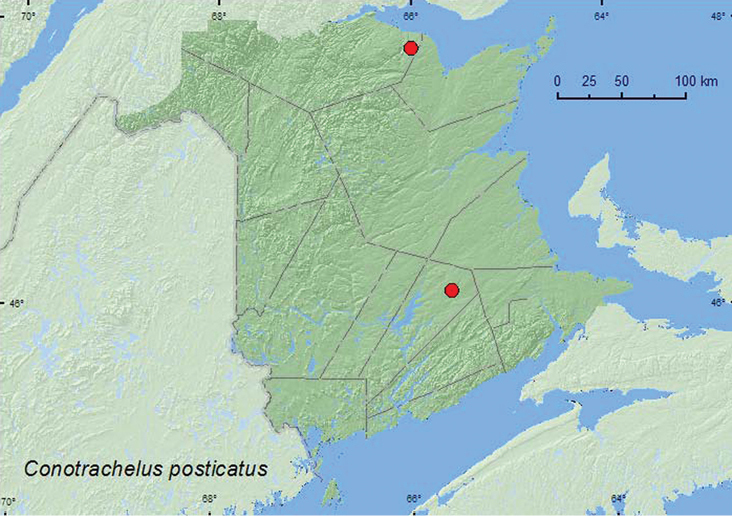
Collection localities in New Brunswick, Canada of *Conotrachelus posticatus*.

##### Collection and habitat data.

*Conotrachelus posticatus*larvae develop in acorns of several oak species (Gibson 1964). Schoof (1942) reported that this species was associated with *Quercus*, *Carya*, *Prunus*, and *Crataegus*. Most specimens from New Brunswick were collected from Lindgren funnel traps in a red oak forest. A few individuals were swept from foliage in the understory. Adults were collected during May, June, July, and August.

##### Distribution in Canada and Alaska.

ON, QC, **NB**, NS (McNamara 1991c; Majka et al. 2007c).

### Tribe Molytini Schönherr, 1823

#### Sthereus ptinoides

(Germar, 1824)

http://species-id.net/wiki/Sthereus_ptinoides

Map 50

##### Material examined.

**New Brunswick, Saint John Co.**, Saint John, Taylors Island, 45.2248°N, 66.1228°W, 28.VIII.2008, R. P. Webster, sea beach, under drift wood (1, RWC).

**Map 50.**
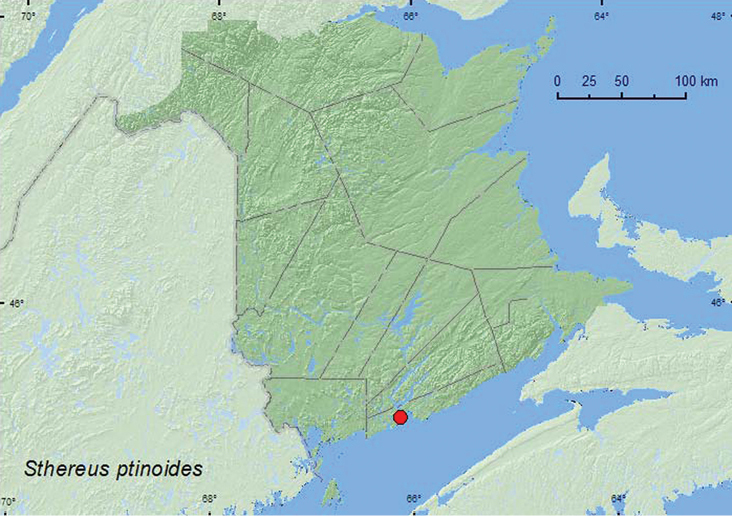
Collection localities in New Brunswick, Canada of *Sthereus ptinoides*.

##### Collection and habitat data.

Anderson (1988) reported this species from under driftwood on beaches of Queen Charlotte Island (official name is now Haida Gwai), British Columbia. The specimen from New Brunswick was likewise found under driftwood on a sea beach. The adult was collected during late August.

##### Distribution in Canada and Alaska.

AK, BC, **NB**, NS, NF (McNamara 1991c).

### Subfamily Scolytinae Latreille, 1804

**Tribe Corythylini LeConte, 1876**

#### Pityophthorus biovalis

Blackman, 1922

http://species-id.net/wiki/Pityophthorus_biovalis

Map 51

##### Material examined.

**New Brunswick, Northumberland Co.**, Neguac, emerged 18.VI.1969, (no collector given), ex. rust galls on *Pinus banksiana* collected on 26.V.1969 (2, AFC).

**Map 51.**
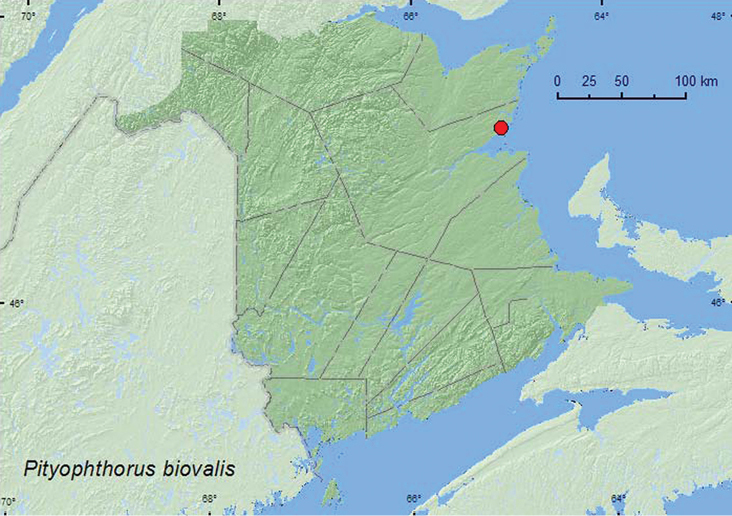
Collection localities in New Brunswick, Canada of *Pityophthorus biovalis*.

##### Collection and habitat data.

Most species of *Pityopthorus* in North America breed in twigs (Drooz 1985). Hosts reported for *Pityophthorus biovalis* include *Picea glauca*, *Picea rubens* and *Pinus strobus* (Wood 1982). The specimens from New Brunswick were reared from rust galls on jack pine (*Pinus banksiana* Lamb.).

##### Distribution in Canada and Alaska.

ON, **NB**, NS (McNamara 1991d).

#### Pseudopityophthorus minutissimus

(Zimmermann, 1868)

http://species-id.net/wiki/Pseudopityophthorus_minutissimus

Map 52

##### Material examined.

**New Brunswick, Carleton Co.**, Jackson Falls, Bell Forest, 46.2200°N, 67.7231°W, 20–26.V.2009, R. Webster & M.-A. Giguère, mature hardwood forest, Lindgren funnel trap (1, AFC). **Queens Co.**, Cranberry Lake P.N.A., 46.1125°N, 65.6075°W, 12–21.V.2009, 27.V-5.VI.2009, 5–11.VI.2009, R. Webster & M.-A. Giguère, mature red oak forest, Lindgren funnel traps (6, AFC, RWC).

**Map 52.**
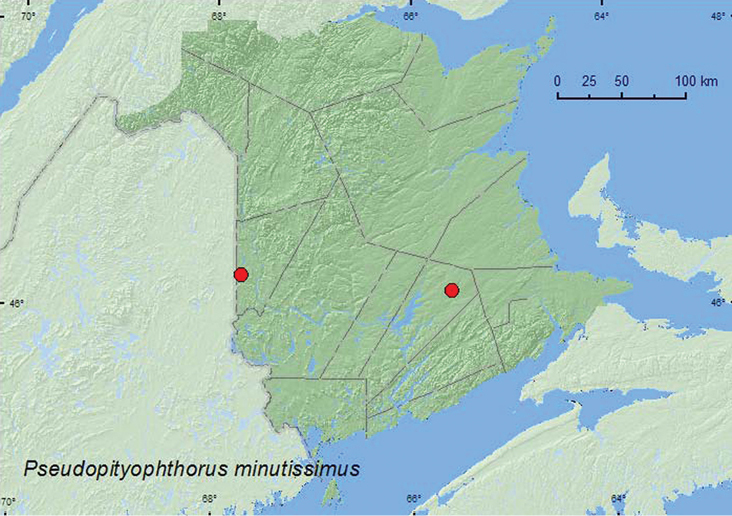
Collection localities in New Brunswick, Canada of *Pseudopityopthorus minutissimus*.

##### Collection and habitat data.

Hosts include various *Quercus* spp. (Bright 1976; Wood 1982). Specimens from New Brunswick were captured during May and June in Lindgren funnel traps in a hardwood forest and an old red oak forest.

##### Distribution in Canada and Alaska.

ON, QC, **NB**, NS (Bright 1976; McNamara 1991d; Majka et al. 2007c).

### Tribe Dryocoetini Lindemann, 1877

#### Dryocoetes caryi

Hopkins, 1915**

http://species-id.net/wiki/Dryocoetes_caryi

Map 53

##### Material examined.

**New Brunswick, Restigouche, Co.**, Dionne Brook P.N.A., 47.9064°N, 68.3441°W, 27.VI–14.VII.2011, M. Roy & V. Webster, old-growth balsam fir and white spruce forest, Lindgren funnel traps (5, AFC, RWC).

**Nova Scotia, Halifax Co.**, McNabs Island, 44.612°N, 63.516°W,, 9.V.2006, Price / Brawn, Lindgren funnel traps, *Ips* lure (4, AFC); same data but 5.VII.2006, Sweeney/Price, Lindgren funnel traps, *Ips* lure (1, AFC).

**Map 53.**
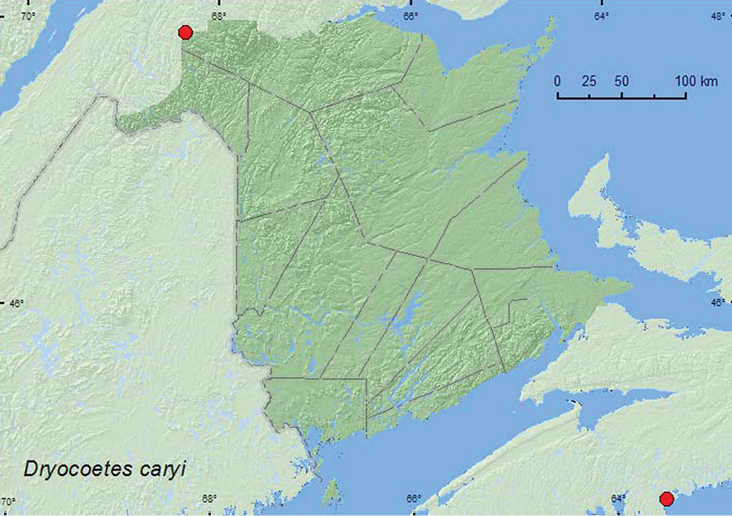
Collection localities in New Brunswick and Nova Scotia, Canada of *Dryocoetes caryi*.

##### Collection and habitat data.

Hosts of this rare species in eastern Canada include suppressed *Picea glauca* and *Pinus rubra* (Bright 1976; Wood 1982). Specimens from New Brunswick and Nova Scotia were captured in Lindgren funnel traps in conifer forests.

##### Distribution in Canada and Alaska.

AK, BC, AB, QC, **NB, NS** (Bright 1976; McNamara 1991d).

### Tribe Hylastini LeConte, 1876

#### Hylastes opacus

Erichson, 1836**

http://species-id.net/wiki/Hylastes_opacus

Map 54

##### Material examined.

**New Brunswick, Queens Co.**, Grand Lake near Scotchtown, 45.8762°N, 66.1816°W, 25.VI.2006, R. P. Webster, red oak and maple forest near lakeshore, in litter near vernal pond (1, RWC). **York Co.**, 15 km W of Tracy off Rt. 645, 45.6837°N, 66.8809°W, 10.VI.2007, R. P. Webster, old red pine forest, underside of red pine log, under bark (1, RWC); 15 km W of Tracy off Rt. 645, 45.6848°N, 66.8821°W, 25.IV–4.V.2009, 11–19.V.2009, 19–25.V.2009, 1–8.VI.2009, R. Webster & M.-A. Giguère, old red pine forest, Lindgren funnel traps (10, AFC, RWC); 14 km WSW of Tracy, S of Rt. 645, 45.6741°N, 66.8661°W, 28.IV–10.V.2010, R. Webster & C. MacKay, old mixed forest with red and white spruce, red and white pine, balsam fir, eastern white cedar, red maple, and *Populus* sp., Lindgren funnel trap (1, AFC).

**Map 54.**
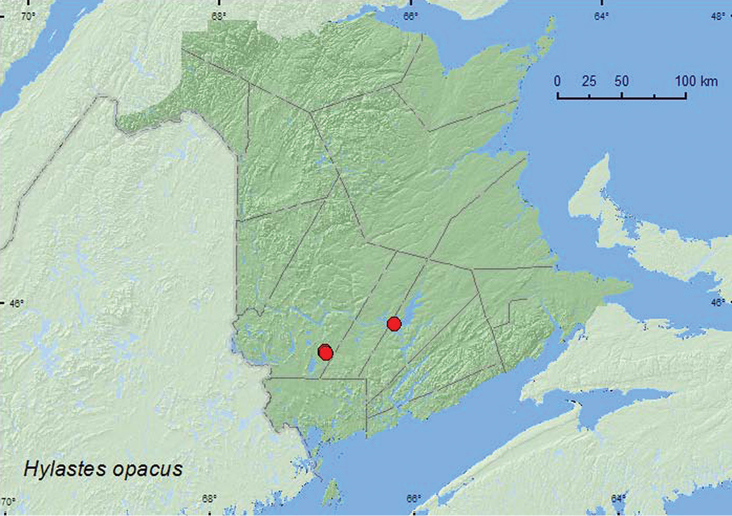
Collection localities in New Brunswick, Canada of *Hylastes opacus*.

##### Collection and habitat data.

Most adults of this adventive species were captured in Lindgren funnel traps in an old red pine and old mixed forest with red pine. One adult was collected from under bark on the underside of a red pine log. Adults were captured in April, May, and June. Bright and Skidmore (1997) reported various species of *Pinus* and *Larix* as hosts for this Palaearctic species where it breeds in stumps and roots of dead and dying trees (Hoebeke 1994).

##### Distribution in Canada and Alaska.

ON, QC, **NB** (Bright and Skidmore 1997).

#### Scierus annectans

LeConte

http://species-id.net/wiki/Scierus_annectans

Map 55

##### Material examined.

**Additional New Brunswick records, Restigouche, Co.**, Dionne Brook P.N.A., 47.9030°N, 68.3503°W, 31.V–15.VI.2011, M. Roy & V. Webster, old-growth northern hardwood forest, Lindgren funnel traps (2, NBM, RWC); same locality and collectors but 47.9064°N, 68.3441°W, 31.V–15.VI.2011, old-growth white spruce and balsam fir forest, Lindgren funnel traps (10, AFC, NBM, RWC).

**Map 55.**
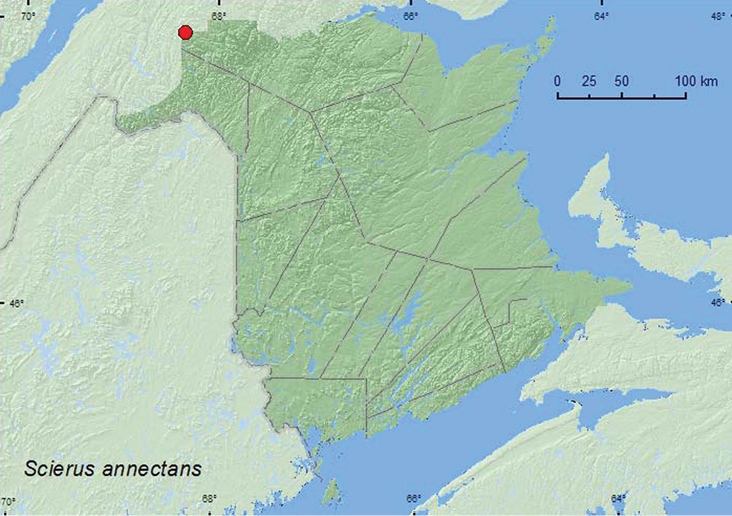
Collection localities in New Brunswick, Canada of *Scierus annectans*.

##### Collection and habitat data.

This species was captured during June in Lindgren funnel traps in an old-growth northern hardwood forest and an old-growth white spruce and balsam fir forest. Hosts in eastern Canada include *Picea glauca* and other *Picea* spp. (Wood and Bright 1992).

##### Distribution in Canada and Alaska.

AK, NT, BC, AB, ON, QC, NB, NS, NF (Bright 1976; McNamara 1991d; Wood and Bright 1992). Although Wood and Bright (1992) reported this species as occurring in New Brunswick, Majka et al. (2007b) did not list this species for the province. The above records confirm the presence of this species in New Brunswick.

### Tribe Hylesinini Erichson, 1836

#### Hylesinus aculeatus

Say, 1824

http://species-id.net/wiki/Hylesinus_aculeatus

Map 56

##### Material examined.

**New Brunswick, Carleton Co.**, Jackson Falls, Bell Forest, 46.2152°N, 67.7190°W, 11.V.2005, 1.VI.2005, R. Webster & M.-A. Giguère, river margin forest with butternut, collected with aerial net during late afternoon flight, (5, RWC); same locality and collector but 46.2200°N, 67.7231°W, 28.IV–9.V.2009, 9–14.V.2009, 14–20.V.2009, 20–26.V.2009, mature hardwood forest, Lindgren funnel traps (7, AFC, RWC). **Kings Co.**, Grays Mills, 1.VI.1921, R. P. G., (1, AFC). **Queens Co.**, Grand Lake Meadows P.N.A., 45.8227°N, 66.1209°W, 4–19.V.2010, R. Webster & C. MacKay, old silver maple forest with green ash and seasonally flooded marsh, Lindgren funnel traps (2, AFC). **York Co.**, South Tweedside, 25.X.1977, (no collector given), camp window (20, AFC); Fredericton, 24.VIII.1978 (emergence date), (no collector given), ex. *Fraxinus americana* (9, AFC); Charters Settlement, 45.8395°N, 66.7391°W, 10.V.2007, 6.V.2008, 4.IV.2010, R. P. Webster, mixed forest, collected with aerial net during late afternoon flights (3, RWC); 15 km W of Tracy off Rt. 645, 45.6848°N, 66.8821°W, 19–25.V.2009, R. Webster & M.-A. Giguère, old red pine forest, Lindgren funnel trap (1, AFC).

**Map 56.**
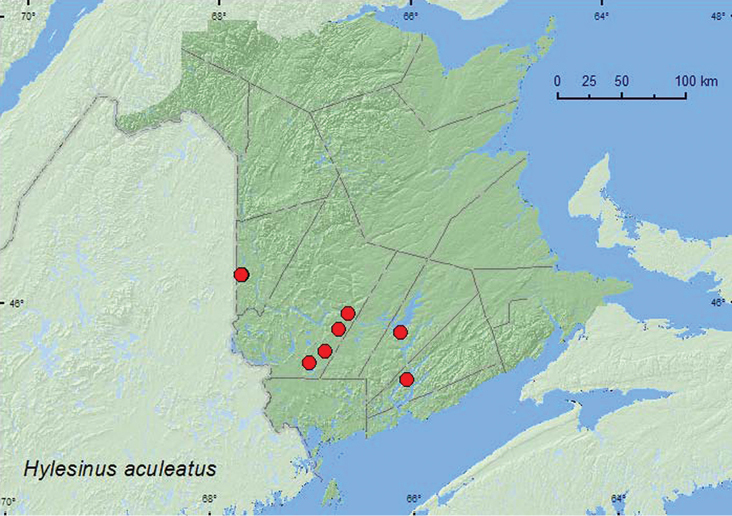
Collection localities in New Brunswick, Canada of *Hylesinus aculeatus*.

##### Collection and habitat data.

Hosts include various species of *Fraxinus* (Wood 1982). Adults from New Brunswick were captured in Lindgren funnel traps in river margin floodplain forests with butternut, white ash, and black ash (*Fraxinus nigra* Marsh.), silver maple forest, hardwood forests with American beech and sugar maple, mixed forests, and a red pine forest. *Fraxinus* was present at all these sites. Adults were also reared from *Fraxinus americana*. This species was collected during April, May, and early June (most during May).

##### Distribution in Canada and Alaska.

SK, MB, ON, QC, **NB**, NS (Bright 1976; McNamara 1991d).

### Tribe Hylurgini Gistel, 1848

#### Xylechinus americanus

Blackman, 1922

http://species-id.net/wiki/Xylechinus_americanus

Map 57

##### Material examined.

**New Brunswick, Restigouche Co.**, Dionne Brook P.N.A., 47.9064°N, 68.3441°W, 31.V–15.VI.2011, M. Roy & V. Webster, old-growth white spruce and balsam fir forest, Lindgren funnel traps (4, NBM, RWC). **York Co.**, 15 km W of Tracy off Rt. 645, 45.6848°N, 66.8821°W, 1–6.VI.2009, R. Webster & M.-A. Giguère, old red pine forest, Lindgren funnel trap (1, RWC); 14 km WSW of Tracy, S of Rt. 645, 45.6741°N, 66.8661°W, 10–26.V.2010, R. Webster & C. MacKay, old mixed forest with red and white spruce, red and white pine, balsam fir, eastern white cedar, red maple, and *Populus* sp., Lindgren funnel traps (3, AFC, RWC).

**Map 57.**
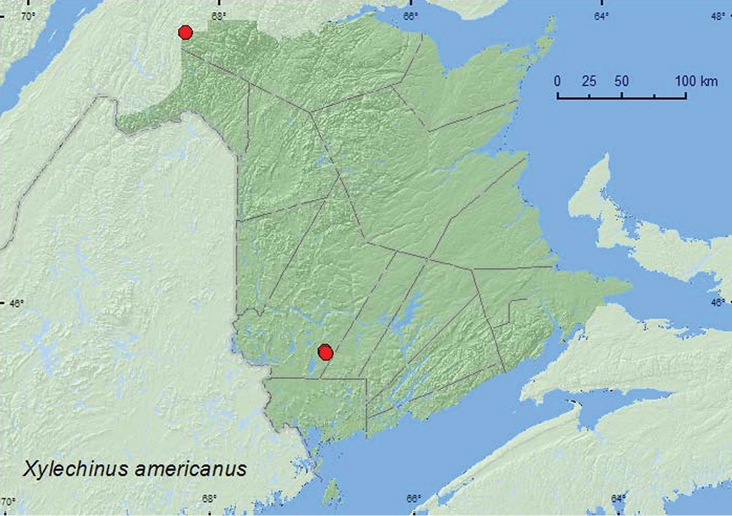
Collection localities in New Brunswick, Canada of *Xylechinus americanus*.

##### Collection and habitat data.

Hosts include *Picea* spp. and *Pinus* spp. (Wood 1982). Adults were captured during May and June in Lindgren funnel traps in an old-growth red pine forest, an old mixed forest, and an old-growth white spruce and balsam fir forest (boreal forest).

##### Distribution in Canada and Alaska.

ON, QC, **NB**, NS (Bright 1976; McNamara 1991d).

### Tribe Ipini Bedel, 1888

#### Ips pini

(Say, 1826 )

http://species-id.net/wiki/Ips_pini

Map 58

##### Material examined.

**Prince Edward Island, Kings Co.**, Goose River, 27.VI.2000, 24.VII.2000, G. Smith, Lindgren funnel trap, *Ips pini* lure (86, AFC).

**Map 58.**
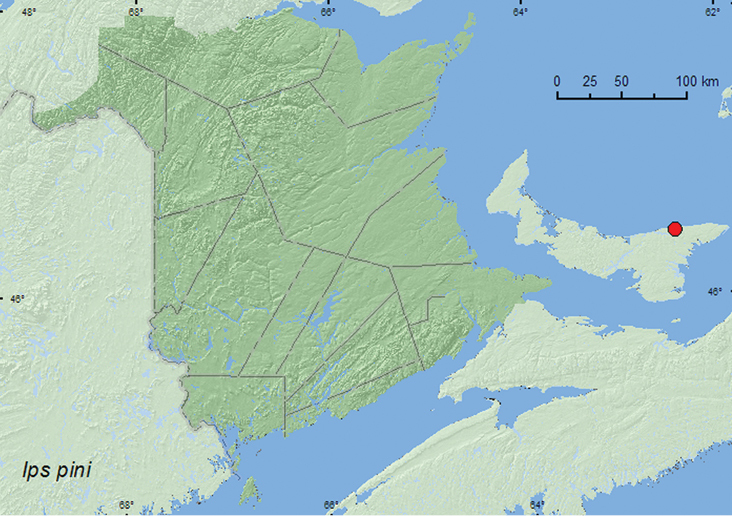
Collection localities in Prince Edward Island, Canada of *Ips pini*.

##### Collection and habitat data.

Host plants of this widespread species include various species of *Pinus* (Wood 1982). Specimens from Prince Edward Island were captured during June and July in Lindgren funnel traps baited with *Ips pini* lures.

##### Distribution in Canada and Alaska.

AK, YK, NT, BC, AB, SK, MB, ON, QC, NB, NS, **PE**, NF (Bright 1976; McNamara 1991d).

#### Orthotomicus latidens

(LeConte, 1874)**

http://species-id.net/wiki/Orthotomicus_latidens

Map 59

##### Material examined.

**New Brunswick, Sunbury Co.**, Acadia Research Forest, 45.9866°N, 66.3841°W, 2–9.VI.2009, R. Webster & M.-A. Giguère, red spruce forest with red maple and balsam fir, Lindgren funnel trap (1, RWC). **York Co.**, Fredericton, 8.VI.1925, 9.VI.1925, L.J. Simpson (5, AFC); 15 km W of Tracy off Rt. 645, 45.6848°N, 66.8821°W, 19–25.V.2009, R. Webster & M.-A. Giguère, old red pine forest, Lindgren funnel trap (1, RWC).

**Nova Scotia, Halifax Co.**, Halifax, Point Pleasant Park, 16.VI.1999, G. Smith, Lindgren funnel trap, *Ips* lure, 99–2-2057–01 (1, AFC).

**Map 59.**
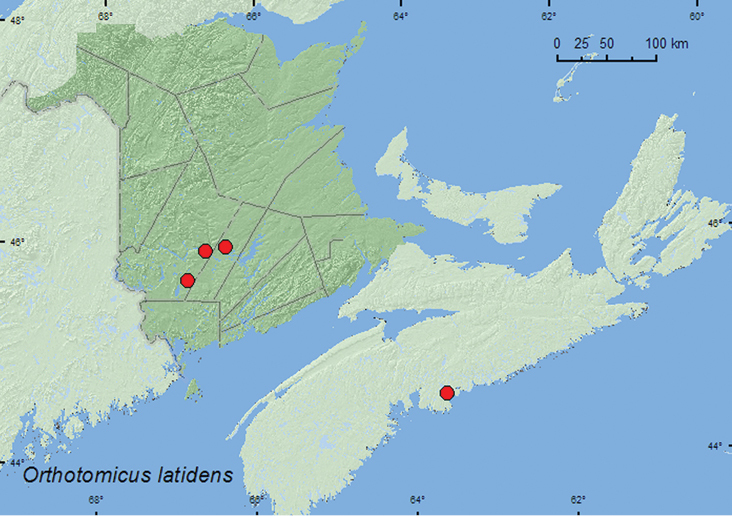
Collection localities in New Brunswick and Nova Scotia, Canada of *Orthotomicus latidens*.

##### Collection and habitat data.

Hosts in eastern Canada include *Pinus* spp. and *Tsuga canadensis* (Wood 1982). In New Brunswick, this species was captured during May and June in Lindgren funnel traps deployed in a red spruce forest and an old red pine forest.

##### Distribution in Canada and Alaska.

YK, BC, AB, SK, ON, QC, **NB, NS** (Bright 1976; McNamara 1991d).

#### Pityogenes plagiatus

(LeConte, 1868)**

http://species-id.net/wiki/Pityogenes_plagiatus

Map 60

##### Material examined.

**New Brunswick, Northumberland Co.**, Meadow Brook Rd., SW of Eel River Bridge, 15.VII.1983, B.A.P., collected from *Pinus resinosa*, 83–2-2371–01 (1, AFC). **York Co.**, 15 km W of Tracy off Rt. 645, 45.6848°N, 66.8821°W, 13.V.2009, R. Webster & M.-A. Giguère, old red pine forest, on small branch of recently fallen red pine (2, AFC, RWC).

**Map 60.**
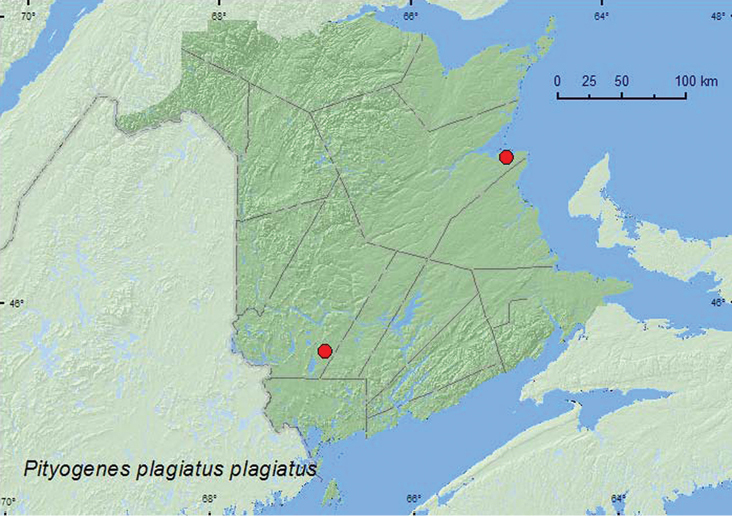
Collection localities in New Brunswick, Canada of *Pityogenes plagiatus*.

##### Collection and habitat data.

Hosts include *Picea* spp., *Pinus banksiana*, and *Pinus resinosa* (Wood 1982). Specimens from New Brunswick were collected during May and July from *Pinus resinosa*, one from a small branch of a recently fallen tree.

##### Distribution in Canada and Alaska.

AB, SK, MB, ON, QC, **NB** (Bright 1976; McNamara 1991d).

### Tribe Xyleborini LeConte, 1876

#### Anisandrus dispar

(Fabricius, 1792)

http://species-id.net/wiki/Anisandrus_dispar

Map 61

##### Material examined.

**New Brunswick, Queens Co.**, Grand Lake Meadows P.N.A., 45.8227°N, 66.1209°W, 19.V–26.VII.2010, R. Webster & C. MacKay, old silver maple forest with green ash and seasonally flooded marsh, Lindgren funnel traps (3, AFC).

**Map 61.**
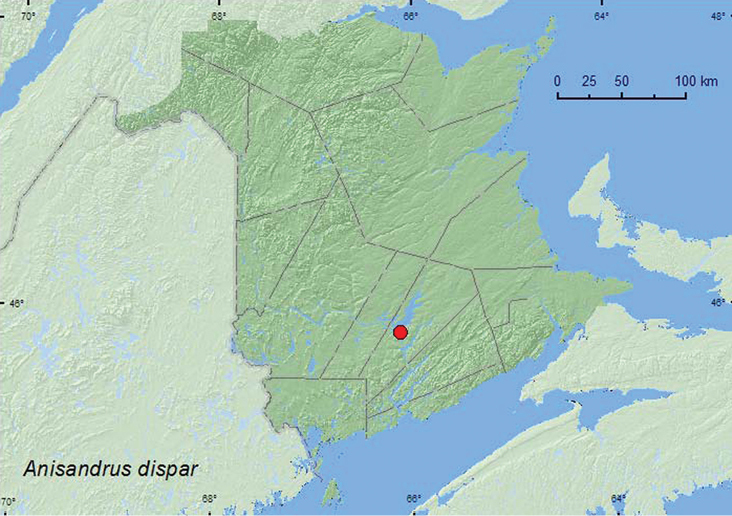
Collection localities in New Brunswick, Canada of *Anisandrus dispar*.

##### Collection and habitat data.

Adults of this adventive species were captured mid May to late July in Lindgren funnel traps in an old silver maple forest.

##### Distribution in Canada and Alaska.

BC, ON, QC, **NB**, NS, NF, PE (Bright 1976; McNamara 1991d; Klimaszewski et al. 2010).

#### Anisandrus obesus

(LeConte, 1868)

http://species-id.net/wiki/Anisandrus_obesus

Map 62

##### Material examined.

**New Brunswick, Carleton Co.**, Jackson Falls, Bell Forest, 46.2200°N, 67.7231°W, 12–19.VI.2008, 19–27.VI.2008, 9–14.V.2009, R. P. Webster, mature hardwood forest, Lindgren funnel traps (6, AFC, RWC). **Queens Co.**, Cranberry Lake PNA, 46.1125°N, 65.6075°W, 24.IV–5.V.2009, 5–12.V.2009, R. Webster & M.-A. Giguère, old red oak forest, Lindgren funnel traps (13, AFC, RWC). **Sunbury Co.**, Acadia Forest Experiment Station, 30.VI.1999, (no collector given), pitfall trap survey, collection site 2, Strip (2, AFC); Acadia Research Forest, 45.9866°N, 66.3841°W, 8–13.V.2009, R. Webster & M.-A. Giguère, red spruce forest with red maple and balsam fir, Lindgren funnel traps (10, AFC, RWC). **York Co.**, 15 km W of Tracy off Rt. 645, 45.6848°N, 66.8821°W, 11–19.V.2009, 19–25.V.2009, R. Webster & M.-A. Giguère, old red pine forest, Lindgren funnel traps (7, AFC, RWC).

**Map 62.**
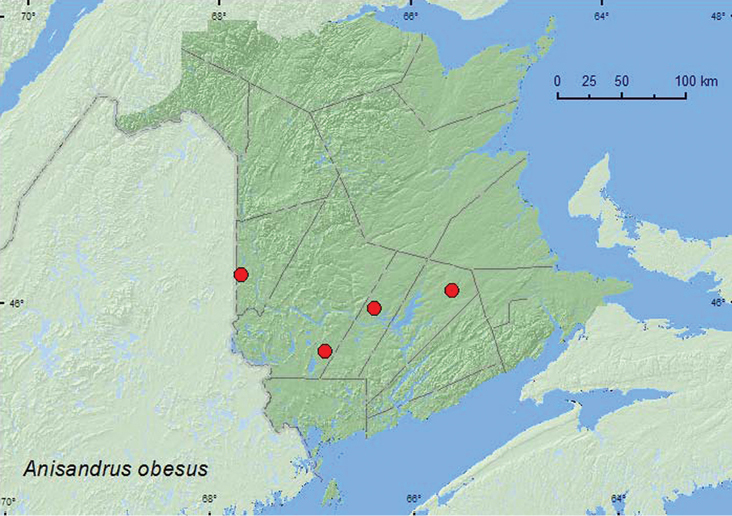
Collection localities in New Brunswick, Canada of *Anisandrus obesus*.

##### Collection and habitat data.

Hosts of this species include *Fagus grandifolia*, *Populus tremuloides*, and *Quercus* spp. (Bright 1976; Wood 1982). In New Brunswick, most adults were captured during May and June in Lindgren funnel traps in hardwood forests, red oak forests, red spruce forests with red maple, and an old red pine forest.

##### Distribution in Canada and Alaska.

ON, QC, **NB**, NS (Bright 1976; McNamara 1991d; Majka et al. 2007c).

#### Anisandrus sayi

Hopkins, 1915

http://species-id.net/wiki/Anisandrus_sayi

Map 63

##### Material examined.

**New Brunswick, Carleton Co.**, Jackson Falls, Bell Forest, 46.2200°N, 67.7231°W, 4–12.VI.2008, 12–19.VI.2008, 19–27.VI.2008, 27.VI–5.VII.2008, 12–19.VII.2008, 19–28.VII.2008, R. P. Webster, mature hardwood forest, Lindgren funnel traps (10, AFC, RWC); same locality and forest type but 9–14.V.2009, 14–20.V.2009, R. Webster & M.-A. Giguère, Lindgren funnel traps (11, AFC). **Queens Co.**, Cranberry Lake P.N.A, 46.1125°N, 65.6075°W, 21–27.V.2009, 5–11.VI.2009, R. Webster & M.-A. Giguère, old red oak forest, Lindgren funnel traps (4, AFC); Grand Lake Meadows P.N.A., 45.8227°N, 66.1209°W, 19–31.V.2010, R. Webster & C. MacKay, old silver maple forest with green ash and seasonally flooded marsh, Lindgren funnel traps (numerous specimens collected in EtOH baited traps) (1, AFC). **Restigouche Co.**, Dionne Brook P.N.A., 47.9030°N, 68.3503°W, 31.V–15.VI.2011, M. Roy & V. Webster, old-growth northern hardwood forest, Lindgren funnel traps (2, AFC, NBM). **Sunbury Co.**, Acadia Forest Experiment Station, 30.VI.1999, (no collector given), pitfall trap survey, collection site 1, Control (2, AFC); Acadia Research Forest, 45.9866°N, 66.3841°W, 8–13.V.2009, 13–19.V.2009, 19–25.V.2009, R. Webster & M.-A. Giguère, red spruce forest with red maple and balsam fir, Lindgren funnel traps (4, AFC). **York Co.**, Fredericton, University of New Brunswick Woodlot, 14.V.1964 (emergence date), C. M. D., ex beech bolt collected on 28.VIII.1963, 63–1280–01 (2, AFC); 15 km W of Tracy off Rt. 645, 45.6848°N, 66.8821°W, 11–19.V.2009, 1–8.VI.2009, R. Webster & M.-A. Giguère, old red pine forest, Lindgren funnel trap (8, RWC); 14 km WSW of Tracy, S of Rt. 645, 45.6741°N, 66.8661°W, 10–26.V.2010, R. Webster & C. MacKay, old mixed forest with red and white spruce, red and white pine, balsam fir, eastern white cedar, red maple, and *Populus* sp., Lindgren funnel trap (1, AFC).

**Map 63.**
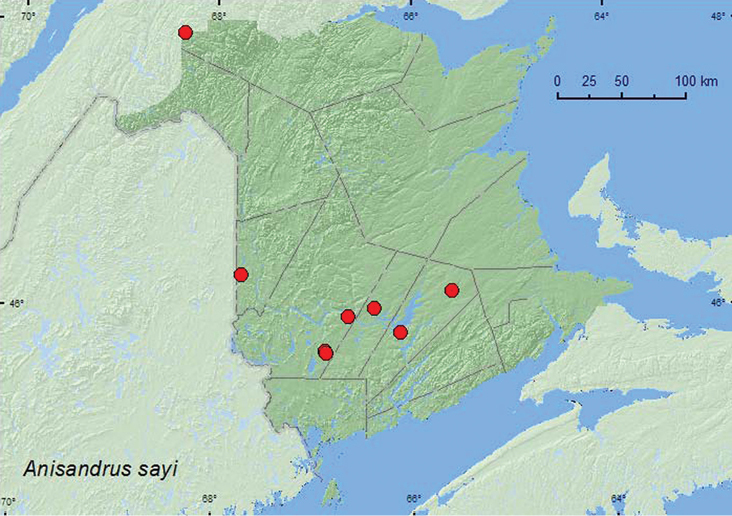
Collection localities in New Brunswick, Canada of *Anisandrus sayi*.

##### Collection and habitat data.

Hosts of this species include various hardwood species (Bright 1976; Wood 1982). In New Brunswick, most adults were captured in Lindgren funnel traps during May, June, and July in hardwood forests, red oak forests, red spruce forests with red maple, and an old red pine forest. Numerous adults were collected in ethanol-baited Lindgren funnel traps in an old silver maple forest. Some adults were also reared from a beech bolt.

##### Distribution in Canada and Alaska.

ON, QC, **NB**, NS (Bright 1976; McNamara 1991d; Majka et al. 2007c).

#### Xyleborinus saxesenii

(Ratzeburg, 1837)

http://species-id.net/wiki/Xyleborinus_saxesenii

Map 64

##### Material examined.

**New Brunswick, Carleton Co.**, Jackson Falls, Bell Forest, 46.2200°N, 67.7231°W, 16–21.VI.2009, R. Webster & M.-A. Giguère, mature hardwood forest, Lindgren funnel trap (1, RWC). **Queens Co.**, Cranberry Lake P.N.A., 46.1125°N, 65.6075°W, 12–21.V.2009, 5–11.VI.2009, 11–18.VI.2009, 25.VI-1.VII.2009, R. Webster & M.-A. Giguère, mature red oak forest, Lindgren funnel traps (6, RWC). **Sunbury Co.**, Acadia Research Forest, 45.9866°N, 66.3841°W, 13–19.V.2009, R. Webster & M.-A. Giguère, red spruce forest with red maple and balsam fir, Lindgren funnel trap (1, AFC). **York Co.**, 15 km W of Tracy off Rt. 645, 45.6848°N, 66.8821°W, 15–21.VI.2009, R. Webster & M.-A. Giguère, old red pine forest, Lindgren funnel traps (4, AFC, RWC).

**Map 64.**
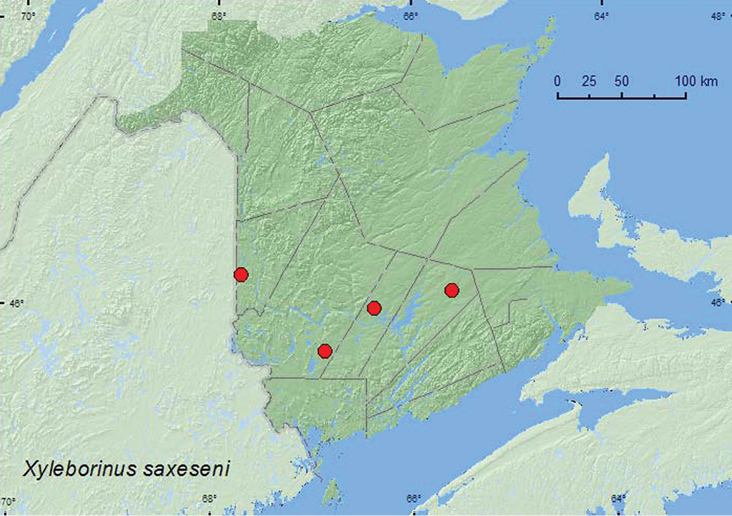
Collection localities in New Brunswick, Canada of *Xyleborinus saxesenii*.

##### Collection and habitat data.

Bright (1976) noted that this adventive species attacks large, dying, deciduous trees and also *Pinus* and *Tsuga* spp. In New Brunswick, all adults were captured during May and June in Lindgren funnel traps in hardwood forests, an old red oak forest, a red spruce forest, and an old red pine forest.

##### Distribution in Canada and Alaska.

BC, ON, QC, **NB**, NS (Bright 1976; McNamara 1991d; Majka et al. 2007c).

## Supplementary Material

XML Treatment for Eurymycter latifascia

XML Treatment for Ormiscus saltator

XML Treatment for Choragus sayi

XML Treatment for Coelocephalapion emaciipes

XML Treatment for Neapion frosti

XML Treatment for Podapion gallicola

XML Treatment for Trichapion porcatum

XML Treatment for Sitophilus oryzae

XML Treatment for Sphenophorus parvulus

XML Treatment for Sphenophorus zeae

XML Treatment for Notiodes ovalis

XML Treatment for Onychylis nigrirostris

XML Treatment for Tanysphrus lemnae

XML Treatment for Anthonomus haematopus

XML Treatment for Anthonomus subfasciatus

XML Treatment for Curculio obtusus

XML Treatment for Ellescus ephippiatus

XML Treatment for Dorytomus frostii

XML Treatment for Dorytomus laticollis

XML Treatment for Dorytomus luridus

XML Treatment for Dorytomus marmoreus

XML Treatment for Cleopomiarus hispidulus

XML Treatment for Piazorhinus pictus

XML Treatment for Bagous obliquus

XML Treatment for Bagous planatus

XML Treatment for Cylindridia prolixa

XML Treatment for Odontocorynus salebrosus

XML Treatment for Plesiobaris disjuncta

XML Treatment for Ceutorhynchus obstrictus

XML Treatment for Pelenomus sulcicollis

XML Treatment for Lechriops oculata

XML Treatment for Cylindrocopturus longulus

XML Treatment for Cossonus americanus

XML Treatment for Stenoscelis brevis

XML Treatment for Himatium errans

XML Treatment for Phloeophagus apionides

XML Treatment for Phloeophagus canadensis

XML Treatment for Phloeophagus minor

XML Treatment for Listronotus deceptus

XML Treatment for Listronotus lutulentus

XML Treatment for Listronotus oregonensis

XML Treatment for Hypera compta

XML Treatment for Lixus rubellus

XML Treatment for Magdalis alutacea

XML Treatment for Magdalis barbita

XML Treatment for Magdalis hispoides

XML Treatment for Magdalis perforata

XML Treatment for Conotrachelus juglandis

XML Treatment for Conotrachelus posticatus

XML Treatment for Sthereus ptinoides

XML Treatment for Pityophthorus biovalis

XML Treatment for Pseudopityophthorus minutissimus

XML Treatment for Dryocoetes caryi

XML Treatment for Hylastes opacus

XML Treatment for Scierus annectans

XML Treatment for Hylesinus aculeatus

XML Treatment for Xylechinus americanus

XML Treatment for Ips pini

XML Treatment for Orthotomicus latidens

XML Treatment for Pityogenes plagiatus

XML Treatment for Anisandrus dispar

XML Treatment for Anisandrus obesus

XML Treatment for Anisandrus sayi

XML Treatment for Xyleborinus saxesenii
